# Microarray analysis of differential gene expression profiles in blood cells of naturally BLV-infected and uninfected Holstein–Friesian cows

**DOI:** 10.1007/s11033-016-4088-6

**Published:** 2016-11-03

**Authors:** P. Brym, S. Kamiński

**Affiliations:** Department of Animal Genetics, University of Warmia and Mazury in Olsztyn, Oczapowskiego 5, 10-719 Olsztyn, Poland

**Keywords:** BLV, Cattle, Host response, Gene expression, Microarrays

## Abstract

The aim of the present study was to examine gene expression changes in response to bovine leukemia virus (BLV) infection, in an effort to determine genes that take a part in molecular events leading to persistent lymphocytosis (PL), and to better define genes involved in host response to BLV infection. Using bovine 70-mer oligonucleotide spotted microarrays (BLOPlus) and qRT-PCR validation, we studied global gene expression profiles in blood cells in vivo of 12 naturally BLV-infected Polish Holstein cows, and 12 BLV non-infected controls of the same breed and reared in herds with high BLV seroprevalence. With an arbitrary cut-off value of 1.5-fold change in gene expression, we identified the down-regulation of 212 genes (M value ≤−0.585) and the up-regulation of 158 genes (M value of ≥0.585) at 1% false discovery rate in BLV-positive animals in comparison to the BLV-negative group. The gene set enrichment analysis demonstrated that the differentially expressed (DE) genes could be classified to diverse biological processes, including immune response of host blood cells. Interestingly, our data indicated the potential involvement of the innate immunity, including complement system activation, NK-cell cytotoxicity and TREM-1 signaling, during the BLV-induced pathogenesis. We showed the occurrence of numerous regulatory processes that are targeted by BLV-infection. We also suggest that a complex network of interrelated pathways is disturbed, causing the interruption of the control of B-cell proliferation and programmed cell death.

**Electronic supplementary material:**

The online version of this article (doi:10.1007/s11033-016-4088-6) contains supplementary material, which is available to authorized users.

## Introduction

Enzootic bovine leukosis (EBL) is the most prevalent, lethal neoplastic disease of cattle, causing considerable economic losses worldwide. The etiological agent of EBL is a B-lymphocytotropic deltaretrovirus, named BLV (Bovine Leukemia Virus), which infects cattle and has distinct clinical outcomes. Most cattle infected by BLV remain clinically silent in an aluekemic state, approximately 30% of infected animals develop a persistent lymphocytosis (PL) which is a benign polyclonal proliferation of B cells, and finally, lethal lymphoma or lymphosarcoma appears in less than 5% of infected animals, after a long latency period [40]. After infection, BLV distributes in the host as integrated proviral DNA, spreading via the cell divisions of the infected leukocytes [20]. The course of leukemogenesis caused by BLV, likewise the mechanisms underlying the aspect of host resistance/susceptibility to BLV infection and disease progression, are intricate and obscured. Although it is not entirely clear, it seems likely that the subtle balance between viral gene expression and efficient host immune response plays a decisive role in the outcome of BLV infection. Furthermore, tumor development in EBL is preceded by an accumulation of chromosomal aberrations and mutations in oncogenes e.g. the p53 gene [8, 18].

An apparent puzzle of BLV-induced leukemogenesis is the fact that in vivo BLV is quiescent in the majority of infected cells, however, vigorous humoral immune response has been elicited in BLV-infected cattle, indicating that BLV is not completely silent. Furthermore, the latent state is immediately reversed when infected lymphocytes are subjected to transient ex vivo cell culture. The potential repression mechanism of viral expression involves a poorly defined plasma blocking factor (PBF), related to fibronectin and inhibited by a platelet lysate [11, 13, 50].

A number of reports suggest that the multifunctional viral accessory protein Tax is the major transforming protein of BLV (reviewed in [13]). The ability of this viral transactivator to both transcriptionally regulate cellular gene expression and directly interact with cellular proteins provides the basis for leukemogenesis [22]. Microarray transcription profiling of ovine immortalized Clone2 B-cell line and peripheral blood-derived ovine B cells cultured in vitro [22] and human HeLa cells [4] transfected with Tax_BLV_ constructs revealed that in vitro Tax was an efficient modulator of host gene transcription. A vast list of Tax-responsive genes contained, among others, genes encoding for cytokines, immune modulators, transcription factors, genes associated with cell cycle, DNA repair and apoptosis, signaling factors and adhesion molecules which could be involved in BLV-induced pathogenesis. It is not yet known whether similar changes in host gene expression occur in vivo during EBL progression in naturally infected cattle.

Evidence to date suggests that host genetic factors may play a role at different stages of infection with BLV: from initial infection to the likelihood of developing PL, through to the fatal lymphoma [14]. It was proved that the resistance and susceptibility to PL development was linked with the MHC class II BoLA-DRB3 gene [26]. The resistance occurs to be dependent upon the presence of polar motif ER at positions 70–71 within a highly polymorphic region encoding the putative peptide binding domain [52]. Recently, one of the alleles containing the ER motif, designated as BoLA–DRB3.2*0902, was shown to be significantly associated with genetic resistance to PL and with a low proviral load profile (LPL) [20]. It was proposed that animals presenting LPL profile and harboring the BoLA–DRB3.2*0902 allele might be unable to transmit BLV in herd conditions, and this suggestion underlay a marker-assisted selection scheme for the simultaneous eradication of BLV and improvement of milk production traits implemented in Argentina [11]. However, the extent of variation in LPL profile not associated with the favorable BoLA–DRB3.2*0902 allele [11] implies that genetic resistance to BLV and the outcome of the disease could be under the control of multiple as yet unknown or unidentified genes, each contributing slightly to the phenotype. In addition, polymorphism in the predicted enhancer region of the bovine TNF-α (tumor necrosis factor, allele −824G associated with low transcription activity) was shown to contribute in the progression of BLV-induced lymphoma [23].

As variation in gene expression could be a causative mechanism underlying BLV resistance/susceptibility, the application of gene expression microarray technology enabling the large scale transcriptome profiling of host immune response promises the identification of networks of genes involved in disease pathogenesis, providing a key for understanding and, perhaps in the future, intervening against successful BLV evasion of host biodefenses. The aim of the present study was to examine gene expression changes in response to BLV infection, in an effort to determine genes that take a part in molecular events leading to PL, and to better define genes involved in host response to BLV infection. We focused on fresh whole blood cells, with their transcriptome freezed and captured immediately after bleeding using RNA stabilization solution, without culturing them because most of these cells in vivo usually do not express viral proteins including Tax, in contrast to either cultured PBMCs from BLV-positive animals or BLV-infected cell lines.

## Materials and methods

### Animals, sample collection and BLV diagnostics

All procedures were approved by the local ethics committee of University of Warmia and Mazury in Olsztyn (no. 13/2008/N/T). Blood samples were collected from two dairy herds, kept in a free stall barn system and located in north-eastern and north-western parts of Poland respectively. BLV seroprevalence rates in both herds exceeded 70%. All sampled animals belonged to the Polish Holstein–Friesian breed and were reared and maintained in the infected herd at least 3 years. The blood was taken from jugular vein of lactating cows using Vacuette® Blood Collection Set and evacuated blood collection tubes: Vacuette® Serum, Vacuette® EDTA (Greiner Bio-One) and Vacuette® Tempus™ Blood RNA Tube (Applied Biosystems, Greiner Bio-One). In other to minimize seasonal effects, all the samples were collected within a single month (November, 2012). BLV serological diagnostics was carried out by ELISA technique using Pourquier® ELISA Bovine Leukosis Screening Kit (Institute Pourquier) according to the manufacture’s protocol. Moreover, the diagnosis was validated with a molecular approach based on BLV proviral DNA detection using nested-PCR method [29]. Automated blood cell counting was performed with SF3000 automated hematology analyzer (Sysmex) according to the manufacture’s instructions. Based on serological (ELISA), molecular (nested-PCR proviral detection) and hematological assays, the experimental group was composed of 12 BLV-infected individuals with apparent hematological alterations characteristic for the lymphoproliferative stage of enzootic bovine leukosis (average lymphocyte count × 10^3^/μl, 15.16 ± 10.28) and the control group was composed of 12 BLV free animals with normal hematological blood indices (average lymphocyte count × 10^3^/μl, 3.43 ± 1.22). The difference in average age of the animals in the experimental group (5 years 2 months, SD = 0.81) and of the control group (4 years 8 months, SD = 0.58) was not significant.

### RNA extraction

Blood samples of 3 ml volume were collected into Vacuette® Tempus™ Blood RNA Tube (Applied Biosystems, Greiner Bio-One) containing 6 ml RNA stabilization solution, mixed vigorously, transported on ice within a few hours to the laboratory and then frozen in −20 °C until RNA isolation. To minimize handling time during the RNA extraction procedure, samples were processed in small batches of 4. After thawing for 30 min. at room temperature each sample was poured into 50 ml Falcon conical tube (BD) and 3 ml of 95% molecular biology grade ethanol (Applichem) was added. The mixture was intensively vortexed on high speed for 5 min. and then centrifuged at 5200×*g* for 60 min at 0 °C. The foaming and the supernatant were poured off and the rim of the inverted Falcon tube was blotted on clean absorbent paper for 1 min. 500 µl of lysis solution supplemented with 5 µl of TCEP solution from PerfectPure™ RNA Cell and Tissue (5 Prime) was added and RNA pellet was dissolved by vortexing for 1 min. The lysate was pipetted onto a purification column (5 Prime). All other procedures including successive centrifugations, washing steps, DNase treatment and RNA elution were according to PerfectPure™ RNA Cell & Tissue manual except prolonged to 45 min. On column DNase digestion at room temperature. Purified RNA concentration, purity, and integrity were determined by A_260_, A_280_ and A_230_ measurements using a NanoDrop ND1000 Spectrophotometer (NanoDrop Technologies) and Agilent 2100 Bioanalyzer with Agilent RNA 6000 Nano Kit (Agilent Technologies). The remaining samples were aliquoted and stored at −80 °C until use.

### Oligonucleotide microarray, aRNA synthesis and labelling, microarray experimental design, hybridization, and image acquisition

The bovine microarray platform used was Bovine Long Oligo Extension (BLOPlus), a 10 K spotted 70-mer oligonucleotide microarray (GEO platform accession: GPL9176), manufactured and purchased from Center for Animal Functional Genomics, Michigan State University. Cy3/Cy5 dye (GE Healthcare) labelled, linearly amplified, antisense aRNA was generated from 1 µg of each sample total RNA using Amino Allyl MessageAmp^TM^II aRNA Amplification kit (Ambion, Life Technologies) according to the manufactures’ instructions. The quantity and the quality of the resulting aRNA was analysed by spectrophotometry and and Agilent 2100 Bioanalyzer with Agilent RNA 6000 Nano Kit (Agilent Technologies). The amount of fluorescent dye incorporated into aRNA was measured as its absorption at 550 nm (Cy3) and 650 nm (Cy5) using a NanoDrop ND1000 Spectrophotometer MicroArray built-in module (NanoDrop Technologies). Preliminary microarray dyE−swap experiments were performed in order to optimize the conditions of microarray hybridization, washing and scanning protocol. Afterwards a common reference design was employed for microarray hybridizations, thus all the aRNAs were labelled with Cy5 and co-hybridized with Cy3 labelled common reference pool aRNA. The common reference aRNA pool was set up by mixing equal amounts of total RNA from all samples in the study and pooled RNA was subsequently used for aRNA linear amplification. Twenty-four arrays were hybridized, representing 12 BLV-infected and 12 non-infected cows. For each microarray slide 60 pmol of Cy5 labelled aRNA was combined with 60 pmol of Cy3 labelled common reference aRNA (5–10 μl of total volume) diluted with SlideHyb#1 glass array hybridization buffer (Ambion, Life Technologies) up to 130 μl and denatured at 70 °C for 5 min. Microarray hybridizations were carried out using a Tecan HS400 Pro hybridization station (Tecan) according to following protocol: step 1 (wash) −70 °C, runs 1, wash time 10 s, soak time 20 s; step 2 (sample injection) −70 °C; step 3 (hybridization) −42 °C/6 h, 35 °C/6 h, 30 °C/6 h with medium agitation frequency; step 4 (wash) 37 °C, runs 5, wash time 10 s, soak time 20 s (2 × SSC, 0.1% SDS) (Ambion, Life Technologies), 25 °C, runs 5, wash time 10 s, soak time 20 s (0.2 × SSC, 0.1% SDS), 25 °C, runs 5, wash time 10 s, soak time 20 s (0.2 × SSC); step 5 (N_2_ slide drying) 25 °C for 2 min. Immediately afterwards microarrays were scanned using a ProScanArray device and ScanArray Express image acquisition and analysis software (Perkin-Elmer).

### Microarray data processing, normalization and analysis

The linear models for microarray data (LIMMA, [45]) software was used to identify differential gene expression. Background correction was performed and within microarray print-tip LOWESS (locally weighted scatterplot smoothing) normalization was employed. No between array normalization was needed (data not shown). For each gene spot, t-statistic, B-statistic, M value (log_2_ [Cy5/Cy3]), A value (1/2 [log_2_ Cy5 + log_2_ Cy3]) were calculated. Probability p values were corrected for multiple testing using the false discovery rate (FDR) [5]. An arbitrary fold change of FC ≥1.5 (represented by −0.585 ≤ M value ≥ 0.585) and FDR-adjusted (adj. p ≤ 0.01) significantly differentially expressed (DE) genes were subjected to meta-analysis using a web-based GeneGo MetaCore™ analytical suite (ver. 5.3, GeneGo, Thomson Reuters). The uploaded list of DE gene IDs was submitted to gene ontology (GO) over-representation analysis including functional categories: GO Processes, GO Molecular Functions, GO Localizations, GeneGo Process Networks and GeneGo Diseases. The in silico predicted functional protein networks were generated using the Analyze Networks (AN) build-in algorithm. Additionally, a list of transcription factors related to the expression regulation of DE genes was conceived by AN Transcription Regulation algorithm.

### Validation of microarray results by real-time qRT-PCR

The validation of gene expression data was performed by qRT-PCR for 14 genes (up-regulated, down-regulated and with no regulation according to microarray data) using a LightCycler® LC 480 II system (Roche). For each target gene a total of 24 experimental cDNA samples in duplicate (from 12 BLV+ and 12 BLV− individuals) and a triplicate of no template control (NTC, molecular grade water) were amplified. All procedures including cDNA synthesis, primers design, PCR reaction set up, cycling conditions, melting curve analysis, amplicon’s size and specificity control, standard curve preparation, PCR efficiency (E) and Error value calculation and quantification cycle (Cq) determination were performed as in [6].The primers and the target amplicons information are presented in Table 4. For qRT-PCR data normalization, a pair of RPLP0 and UCHL5 was employed, as the most stable reference genes for this experiment, based on a previous determination from a panel of 10 putative reference gene candidates [6]. The gene expression differences between BLV-infected and BLV uninfected groups were estimated with Relative Expression Software Tool REST 2009 V2.0.13 [37].

## Results

### Microarray analysis of differentially expressed genes in blood cells of naturally BLV-infected and uninfected Holstein cattle

To better understand the role of the genes involved in the host response to BLV infection and BLV-induced lymphoproliferation and malignant transformation, we performed gene expression profiling of BLV-infected and uninfected Holstein cattle. The applied microarray platform comprised the two-color, 70-mer oligonucleotide spotted Bovine Long Oligo Plus (BLOPlus, 10 K) array and the obtained data was deposited in NCBI Gene Expression Omnibus with accession no. XXXXXX. An analysis of mRNA abundance in blood samples from 12 BLV infected cattle and 12 non-infected controls was performed. The BLV infected group comprised animals ca. 5 years old, seropositive with apparent hematological abnormalities (Table 1). Due to the strict BLV eradication policy in Poland, we were able to blood sample the BLV-positive individuals only once, so we could not officially categorize them as in the persistent lymphocytosis (PL) stage of disease. However, the high total lymphocyte counts (15.16 ± 10.28 × 10^3^/μl, Table 1), high total white blood cell counts (WBC), high titers of antibodies against p24 and gp51 and qualitative estimation of BLV proviral loads by PCR products densitometry analysis [29], (data not shown) indicated that this certainly was the case. The BLV-uninfected control group comprised cows of the same breed, similar age (Table 1), diagnosed as BLV-negative by both serological and molecular approaches. It is of interest to emphasize the fact that all the studied BLV-negative animals were housed for at least 3 years in the same 2 BLV-positive herds as their BLV-infected counterparts. Nevertheless they did not become infected. As resistance and susceptibility are opposite sides of the same coin, it is tempting to treat the BLV-negative group not only as the control group, but also to reverse the microarray scheme and to suspect the BLV-negative animals as constituting some degree of innate resistance to BLV infection. Because of the critical dependence of gene expression measurements on starting RNA quality, we included in our analysis only samples which were marked by high RNA quality indices, and without any significant differences between the compared groups (Table 1).

**Table 1:** Comparison of total lymphocyte counts, age of the animals and RNA quality indices in cows analyzed in the study classified to BLV infected and healthy groups based on ELISA and nested-PCR tests

Parameter	BLV+ N = 12		BLV− N = 12	
	$\bar{x}$	SD	$\bar{x}$	SD
Lymphocytes × 10^3^/µl	15.16^a^	10.28	3.43^a^	1.22
Age (year)	5.23	0.81	4.88	0.58
RNA quality				
A_260_/A_280_	2.08	0.022	2.08	0.027
RIN	9.16	0.59	9.04	0.49
28S/18S	1.97	0.51	1.79	0.33

^a^(p ≤ 0.01) Kolmogorov–Smirnov K-S test, *RIN* RNA integrity number

A total of 24 microarrays were hybridized, scanned and analyzed using a common reference experimental design. With an arbitrary cut-off value of 1.5 fold change (FC) in gene expression, we identified the down-regulation of 212 genes (M value ≤−0.585) and the up-regulation of 158 genes (M value of ≥0.585) at a 1% false discovery rate in BLV-positive animals in comparison to the BLV-negative group. The maximum value of down-regulation (FC = −3.21) was noted for two genes S100A4 (S100 Calcium-Binding Protein A4, formerly known as MTS1 or Metastasin) and CFD (Complement Factor D, formerly known as Adipsin). The most up-regulated gene (FC = 3.18) in blood cells from BLV-positive in comparison to BLV-negative cows was ADRA2A encoding Adrenoceptor Alpha 2A, a member of the G protein-coupled receptor superfamily. Lists of the top 25 down- and up-regulated genes in BLV-infected vs BLV-uninfected groups classified according to decreasing statistical significance of gene expression estimation (B-statistics) are presented in Tables 2 and 3 respectively. The complete lists of 212 and 158 DE genes are presented in the supplemental files (Tables 2S and 3S). Hierarchical clustering HCL heatmap for 370 differentially expressed genes and 24 samples (12 BLV-infected vs 12 BLV non-infected) analyzed in the study is presented in the supplementary Fig. 6S. Assuming gene expression fold changes FC ≥2 and FC ≥1.75 at p value (FDR-adjusted) <0.01 we were able to identify 33 and 70 up-regulated genes and almost twice as many down-regulated genes (64 and 130, respectively) in BLV-infected cattle in comparison to the control group. These values indicated that low (~1.5 fold) and medium (≥1.75 fold) gene expression changes were the most abundant classes, characteristic for BLV-induced progression to the lymphocytotic stage of the disease. The higher number of down-regulated genes than up-regulated ones may reflect the viral latency state, typical for the majority of BLV-infected cells in vivo and/or the evasion of the host’s immune surveillance.

**Table 2:** The list of top 25 down-regulated genes in BLV-infected cattle in comparison to BLV-negative group

Oligo_id	Gene symbol	Gene	Ref. seq	Fold change	p value FDR adj.	B stat.
Bt00001447	CFD	Complement factor d (adipsin)	NM_001034255	−3.21	1.743E−10	22.5
Bt00007539	ITCH	Itchy E3 ubiquitin protein ligase homolog (mouse)	NM_001082428	−2.30	1.722E−07	14.1
Bt00007239	CD63	CD63 molecule	NM_205803	−2.57	1.722E−07	13.9
Bt00000825	AIF1	Allograft inflammatory factor 1	NM_173985	−3.13	2.151E−07	13.6
BLO_ext_00163	F5	Coagulation factor V (proaccelerin, labile factor)	NM_173879	−2.50	2.744E−07	13.2
Bt00005024	S100A4	S100 calcium binding protein A4	NM_174595	−3.21	3.990E−07	12.8
Bt00002294	LGALS1	Lectin, galactosidE−binding, soluble, 1	NM_175782	−2.79	7.355E−07	12.0
Bt00005520	SLC40A1	Solute carrier family 40 (iron-regulated transporter), member 1	NM_001077970	−2.24	1.479E−06	11.2
BLO_ext_00934	SIRPA	Signal-regulatory protein alpha	NM_175788	−2.78	1.921E−06	10.9
Bt00002021	CST3	Cystatin C	NM_174029	−1.83	2.586E−06	10.5
BLO_ext_00656	LGMN	legumain	NM_174101	−2.02	5.908E−06	9.5
Bt00004059	NFAM1	NFAT activating protein with ITAM motif 1	XM_002687969	−2.24	8.704E−06	9.1
BLO_ext_01819	CEBPA	CCAAT/enhancer binding protein (C/EBP), alpha	NM_176784	−2.32	9.307E−06	9.0
Bt00007592	PLA2G7	Phospholipase A2, group VII (platelet-activating factor acetylhydrolase, plasma)	NM_174578	−2.28	9.668E−06	8.9
BLO_ext_00486	CD2	CD2 molecule	NM_001011676	−2.21	9.766E−06	8.8
Bt00003322	STAT4	Signal transducer and activator of transcription 4	NM_001083692	−1.95	9.766E−06	8.8
Bt00002910	TGFBI	Transforming growth factor, beta-induced, 68 kDa	NM_001205402	−2.71	1.164E−05	8.6
Bt00007971	AMICA1	Adhesion molecule, interacts with CXADR antigen 1	NM_001080250	−2.13	1.243E−05	8.5
Bt00001417	PLA2G16	Phospholipase A2, group XVI	NM_001075280	−1.89	1.530E−05	8.3
Bt00003194	MTMR9	Myotubularin related protein 9-like	NM_001046256	−2.26	1.673E−05	8.1
BLO_ext_00190	TYROBP	TYRO protein tyrosine kinase binding protein	NM_174627	−2.54	1.833E−05	8.0
Bt00007151	ANXA3	Annexin A3	NM_001035325	−1.81	1.872E−05	7.9
Bt00007329	LGALS3	Lectin, galactosidE−binding, soluble, 3	NM_001102341	−1.89	2.056E−05	7.8
BLO_ext_01078	CAPN2	Calpain 2, (m/II) large subunit	NM_001103086	−1.83	2.220E−05	7.7
Bt00003792	ITGB7	Integrin, beta 7	NM_001105365	−1.93	2.363E−05	7.6

**Table 3:** The list of top 25 up-regulated genes in BLV-infected cattle in comparison to BLV-negative group

Oligo_id	Gene symbol	Gene	Ref. seq	Fold change	p value FDR adj.	B stat.
Bt00004943	MSH2	MutS homolog 2, colon cancer, nonpolyposis type 1 (*E. coli*)	NM_001034584	2.25	7.54E−09	18.5
Bt00004141	KBTBD8	T cell activation kelch repeat protein	NM_001192696	2.66	1.78E−08	17.3
Bt00006375	ADRA2A	Alpha2A adrenergic receptor	NM_174499	3.18	2.39E−08	16.8
BLO_ext_00185	MS4A1, CD20	MembranE−spanning 4-domains, subfamily A, member 1	NM_001077854	2.95	2.39E−08	16.6
BLO_ext_00550	ADORA2B	Adenosine A2b receptor	NM_001075925	2.38	8.08E−08	15.3
Bt00002242	PPA2	Pyrophosphatase (inorganic) 2	NM_001076396	2.16	1.34E−07	14.7
Bt00000295	CD19	CD19 molecule	NM_001245998	2.12	1.58E−07	14.3
BLO_ext_00374	APEX1	APEX nuclease (multifunctional DNA repair enzyme) 1	NM_176609	2.05	1.58E−07	14.3
Bt00000725	HIF1A	Hypoxia inducible factor 1, alpha subunit (basic helix-loop-helix transcription factor)	NM_174339	2.56	1.72E−07	14.0
BLO_ext_01490	SLC4A10	Solute carrier family 4, sodium bicarbonate transporter-like, member 10	NM_001038128	3.05	2.27E−07	13.5
Bt00006625	CCT5	T-complex protein 1, chaperonin containing TCP1, subunit 5 (epsilon)	NM_001034595	1.75	2.48E−07	13.4
Bt00005595	SH3GLB2	SH3-domain GRB2-like endophilin B2	NM_001076802	2.13	4.43E−07	12.6
Bt00001908	LMO2	LIM domain only 2 (rhombotin-like 1)	NM_001076352	2.16	4.43E−07	12.6
Bt00007026	CCNG1	Cyclin G1	NM_001013364	1.97	6.94E−07	12.1
Bt00003454	BANK1	B-cell scaffold protein with ankyrin repeats 1	XM_002688119	1.78	7.36E−07	12.0
Bt00004115	HADH	Hydroxyacyl-CoA dehydrogenase	NM_001046334	2.15	1.22E−06	11.4
BLO_ext_01117	HSD17B4	Hydroxysteroid (17-beta) dehydrogenase 4	NM_001007809	2.39	1.59E−06	11.1
BLO_ext_00320	DYNLL1	Cytoplasmic dynein light polypeptide 1	NM_001003901	2.02	1.97E−06	10.8
Bt00006382	SIRT5	Sirtuin 5	NM_001034295	1.98	2.41E−06	10.6
Bt00000583	C1QBP	Complement component 1, q subcomponent binding protein (C1QBP), nuclear gene encoding mitochondrial protein	NM_001034527	1.99	3.03E−06	10.3
Bt00002141	GMPS	Guanine monphosphate synthetase	BC111273	1.77	4.24E−06	9.9
Bt00003030	HSD17B10	Hydroxysteroid (17-beta) dehydrogenase 10	NM_174334	1.86	4.61E−06	9.8
Bt00003407	TRMT112	tRNA methyltransferase 11 − 2 homolog (*S. cerevisiae*)	NM_001045981	1.85	4.61E−06	9.8
Bt00004269	SRSF9	Serine/argininE−rich splicing factor 9	NM_001083398	1.77	5.91E−06	9.5
BLO_ext_00630	zfp106	Zinc finger protein 106 homolog (mouse)	XM_002690810	2.08	9.57E−06	8.9

### Validation of the microarray results by real-time qRT-PCR method

**Fig. 1:**
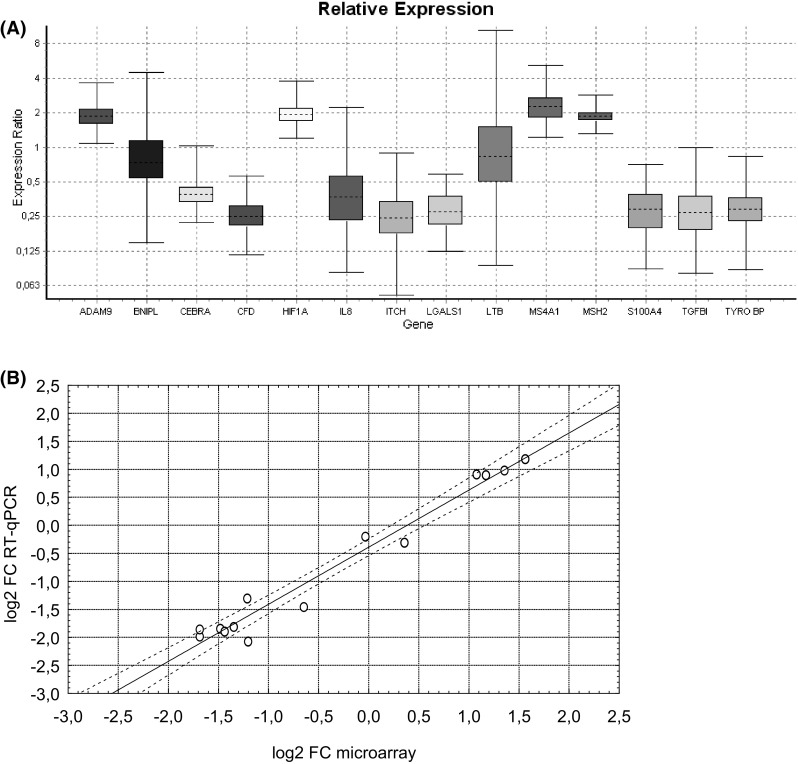
Real-time qRT-PCR validation of microarray results. **a** *Whisker-box* plots of relative expression values for 14 genes in BLV-infected group in comparison to non-infected control. The median of gene expression is depicted by the *dotted line. Whiskers* and *box* show the minimum and maximum values and the interquartile range of observations, respectively. **b** Correlation plot between fold-change (FC) differences revealed by gene expression measurement methods (microarray vs. qRT-PCR). The regression line among the results from different platforms is described by equation: log2FC-qRT-PCR = −0.39 + 1.02 × log2FC-microarray, with Pearson’s correlation coefficient (r) equals 0.98

The accuracy of gene expression estimations from the microarray analysis was validated by the real-time qRT-PCR method. The analysis was performed on blood samples from the same 24 animals used in microarray analysis, although the technical replicate of RNA isolation was used for the reverse transcription reaction. A total of 14 genes were studied including 8 genes down-regulated according to microarray results (i.e.: CFD, ITCH, S100A, LGALS, TGFBI, CXCL8, DAP12, and C/EPB alpha, Table 2) and 4 up-regulated genes (i.e. MS4A1, HIF1A, MSH2 and ADAM9, Table 3) in the BLV-infected group in comparison to the BLV-uninfected animals. Additionally, two genes with no differential expression between the studied groups were randomly selected, i.e. LTB encoding lymphotoxin beta (fold change, FC = −1.02, p value FDR adjusted = 0.929) and BNIPL encoding B-cell lymphoma (BCL2)/adenovirus E1B 19kD interacting protein (fold change, FC = 1.2, p value FDR adjusted = 0.36). For qRT-PCR data normalization, a pair of RPLP0 and UCHL5 gene was employed, based on stable expression in BLV-infected leucocytes which was determined previously [6]. The target amplicons information is presented in Table 4, and their specificity was confirmed by DNA sequencing (data not shown). The relative gene expression measurements between the BLV-infected and the BLV-uninfected groups were estimated with REST 2009 software (Fig. 1a) and indicated significant differences in gene expression levels in all analyzed genes except LTB and BNIPL (p values: 0.46 and 0.11 respectively). The calculated fold changes were log_2_-transformed and compared with log_2_ M-values from microarray analysis. The regression line among the results from different methods is described by the equation: log_2_FC-qRT-PCR = −0.39 + 1.02 × log_2_FC-microarray, with Pearson’s correlation coefficient (r) equaling 0.98 (Fig. 1b). The high value of the correlation coefficient holds out the hope that the rest of the gene expression measurements on the microarray are also valid.

**Table 4:** The genes validated by real-time quantitative reverse transcription PCR (qRT-PCR) method

Gene symbol	Gene name	Function	Primer sequences (forward/reverse) (5′–3′)	Anneal-ing temp. (^o^C)	T_m_	Ampli-con size	Exon junction/intron spanning	E	*Error*	Reference/accession No.
ADAM9	A disintegrin and metalloproteinase domain 9	Zinc protease	F-TGCTGCAGAAGGAAGAGATCACAGA R-CTGAGGCTGCCTGGCTCCGTAA	60	82.2	174	5061	2.02	0.0257	In house design/NM_001192818
MS4A1	MembranE−spanning-4-domains subfamily A member 1	Regulation of B-cell activation and prolifereation	F-ACAGCTGGCACTGTTGAGAATGAA R-CAGCTGACAGGAGAACAACGTTAGC	60	79.2	76	Yes	1.98	0.0058	In house design/NM_001077854
MSH2	MutS homolog 2	DNA mismatch repair	F-ACAGCCTTGGCCAATCAGATACCA R-GCAAGCTCAGCAACATGAATCCCA	62	80.1	140	1735	1.99	0.0125	In house design/NM_001034584
HIF1A	Hypoxia inducible factor 1 alpha subunit	Transcription factor	F-CAGCCACCAGTGATGAATTG R-TGGCACAAGGAGGTTCTTTAG	58	78.9	111	No	1.98	0.0079	[32]/NM_174339
CFD	Adipsin, complement factor D	Component of the alternative complement pathway	F-CATCCCACTCCCGGCCCTACA R-TCCCATCGGCCACGTCCTCCA	58	87.9	126	Yes	1.99	0.0021	In house design/NM_001034255
ITCH	Itchy E3 ubiquitin protein ligase	Control of cell differentiation, inflammation	F-CGACTGCCAGTTGGAGGATTTGC R-AGCTCTTGTAGGGTGGCAGGT	60	83.4	142	Yes	1.96	0.0204	In house design/NM_001082428
S100A4	S100 calcium binding protein A4	Calcium dependent protein binding	F-ACGAGCTCTCCCCAGCACTTCC R-GCAGTTTCATCCGTCCTTTTCCCCA	60	87.0	207	Yes	1.96	0.0081	In house design/NM_174595
CEBPA	CCAAT/Enhancer Binding Protein (C/EBP), alpha	Transcription factor	F-ATCGCGGTGCGCAAGAGCC R-CCCCGCAGCGTGTCCAGTTCG	60	88.2	140	No	1.98	0.0049	In house design/NM_176784
TYROBP	TYRO protein tyrosine kinase binding protein, DAP12	Signal transduction	F-GCTACGGAGGCAGTGACCCGGAAA R-CTCAGGTCGTTGACTGCCCTGCT	60	86.0	152	Yes	2.04	0.0056	In house design/NM_174627
LGALS1	Lectin, galactoside−binding, soluble, 1	Regulation of apoptosis, cell differentiation and proliferation	F-GTTTCAACGCGCATGGGGACG R-AGATGCATACCTCCACGACACTTCC	60	87.5	126	Yes	1.94	0.005	In house design/NM_175782
CXCL8	Interleukin 8	Chemokine	F-GGAAAAGTGGGTGCAGAAGGT R-GGTGGTTTTTTTCTTTTTTCATGGA	55	78.9	82	Yes	1.99	0.0081	In house design/NM_173925
TGFBI	Transforming growth factor, beta-induced, 68 kDa	Cell adhesion	F-CTCTCCAAGGCGACAAGCTGGA R-GTTCCTGAGGTCTGTTGGCTGGAGG	60	86.0	153	Yes	1.95	0.0012	In house design/NM_001205402
BNIPL	BCL2/adenovirus E1B 19kD interacting protein like	Regulation of apoptosis	F-ACAGCCTCCCACCCTATGCCC R-GCTGTGGAGTCAGTCCTGTGCC	58	78.8	105	No	1.99	0.0257	[27]/NM_001079621
LTB	Lymphotoxin beta, TNF superfamily member 3	Cytokine	F-CTACAATAAACCCTCCACGG R-CAGACAGGACATCTCCATCC	60	86.3	117	No	1.92	0.0018	[33]/XM_002697371
UCHL5 reference gene	Ubiquitin carboxyl-terminal hydrolase L5	Deubiquitinat-ing protease	F-ACAAAGACAACTTGCTGAGGAACCC R-GGCAACCTCTGACTGAATAGCACTT	60	78.8	79	Yes	1.96	0.0061	[6]/NM_174481
RPLP0 reference gene	Ribosomal protein large P0	Ribosomal protein, translation	F-CAACCCTGAAGTGCTTGACAT R-AGGCAGATGGATCAGCCA	58	86.3	227	1624	1.95	0.0120	[9]/NM_001012682

E−PCR efficiency calculated using formula E = 10^−1/slope^, *error* value−mean squared error of the single data point fit to the regression line

*T*_*m*_ melting temperature curve peak analysis

### Gene set enrichment analysis of DE gene lists generated from the microarray experiment

To improve the amount and the quality of the ontological information, a total of 370 DE genes identified with the BLOPlus microarray were updated in annotation terms and gene IDs using probe sequence information and BLAST analysis. The list of updated gene IDs and the expression data were input into online meta-analysis software GeneGo MetaCore™ analytical suite (ver. 5.3, GeneGo, Thomson Reuters).

**Fig. 2:**
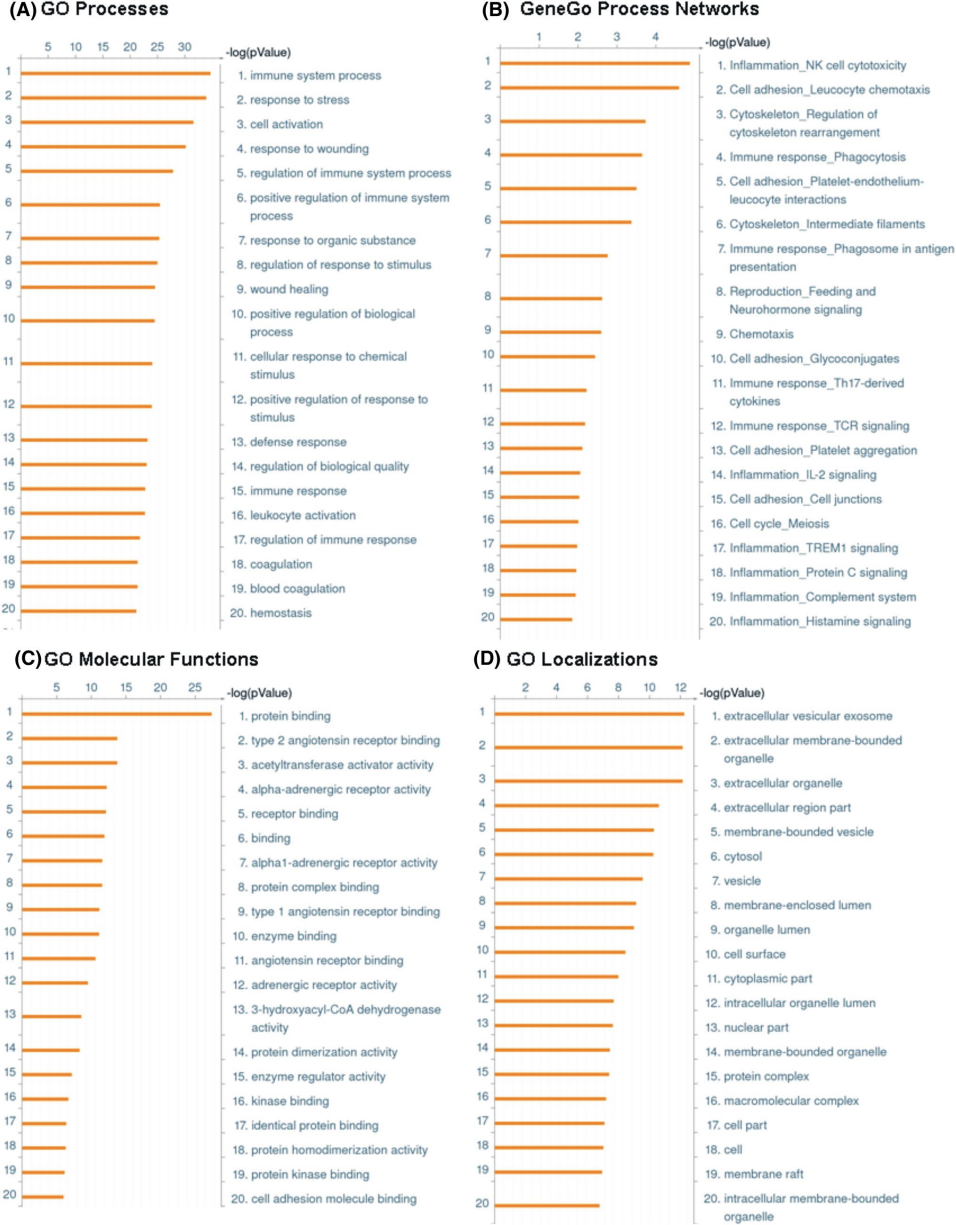
Enrichment analysis of differentially expressed genes identified in this study. Bar length indicates the significance and equals to the negative logarithm of enrichment p value. **a** Top 20 significant biological processes from GO ontology. **b** Top 20 significant process networks from MetaCore ontology. **c** Top 20 significant molecular functions from GO ontology. **d** Top 20 significant localization (cellular compartments) from GO ontology

First, public gene ontology (GO) categories, i.e. GO Processes (biological process), GO Molecular Functions and GO Localizations (cellular component) were assessed, by that disclosing the biological motifs associated with the expression differences observed. The results indicated that the genes with differential expression (DE) could be classified to the numerous gene ontology terms, although with a substantial level of overlapping and redundancy (Fig. 2a, c, d). The enrichment analysis reports, including the top-100 GO terms with calculated p value, FDR and the list of genes involved in each GO term, are presented in the supplementary MS Excel files (Tables 5S, 6S, 7S). Excluding some overlapping terms, we were able to select diverse biological process categories, significantly affected by BLV-infection and the host response, such as: immune system process, response to stress and wounding, regulation of immune response, cell activation, innate immune response, coagulation, regulation of programmed cell death, regulation of metabolic processes, regulation of cell communication, regulation of signal transduction, regulation of cell proliferation, and DNA repair. In addition, the more detailed GeneGo Process Networks Ontology, based on a manual curation database (MetaCore™ ver. 5.3, GeneGo, Thomson Reuters), further delineated the most affected processes and functional networks, among others: inflammation in particular NK-cell cytotoxicity, complement system, IL-2, TREM-1 and protein C signaling; cell adhesion and chemotaxies; immune response in a particular phagosome at antigen presentation, Th-17 derived cytokines, TCR signaling; apoptosis and signal transduction (TGF-beta, GDF, activin and leptin signaling) (Fig. 2b and supplemental MS Excel Table 8S). Among GO molecular functions the most abundant category was represented by protein binding (Fig. 2c). On the other hand, GO Localizations enrichment analysis pointed to the importance of the extracellular region in BLV-induced pathogenesis (Fig. 2d).

**Fig. 3:**
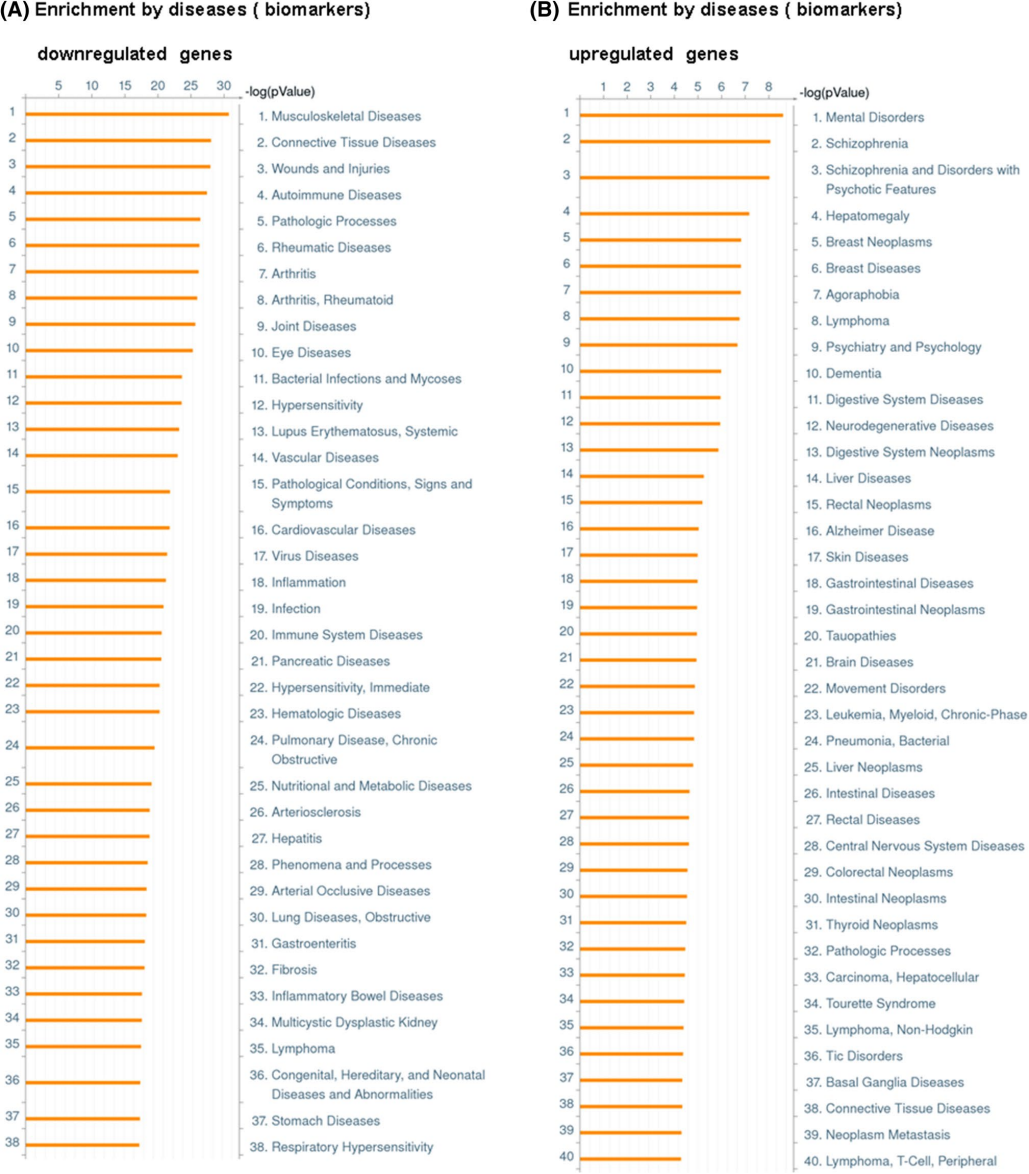
Enrichment analysis of differentially expressed genes identified by disease biomarkers (GeneGO). Bar length indicates the significance and equals to the negative logarithm of enrichment p value. **a** Diseases associated with the genes down-regulated in BLV-infected cattle in comparison to non-infected controls. **b** Diseases associated with the genes up-regulated in BLV-infected cattle in comparison to non-infected controls

Furthermore, the gene set enrichment analysis by GeneGo Disease Biomarkers Ontology linked the genes down-regulated in BLV-positive individuals with, among others: connective tissue diseases, numerous autoimmune diseases, viral diseases and lymphoma. In contrast, the genes with up-regulated expression in PL animals were connected with, as expected lymphoma, non-Hodgkin’s lymphoma, numerous neoplasms, and, unexpectedly schizophrenia and mental disorders (Fig. 3a, b). Although we used in our comparisons animals similar to PL, which is thought to be a benign, preneoplastic stage of the disease, a total of 87 genes from our DE data set was linked with lymphoma and an even greater number of genes were linked with biomarkers of the neoplastic transformation of different organs. It may be suggested that deregulation of not a few but rather multiple genes established the transcriptional foundation of the initial transformation events induced by BLV towards a leukemogenesis. In addition, the observed enrichment of the genes associated with autoimmune diseases, sheds light on a new possible mechanism contributing to a failure of the host immune response against BLV.

**Fig. 4:**
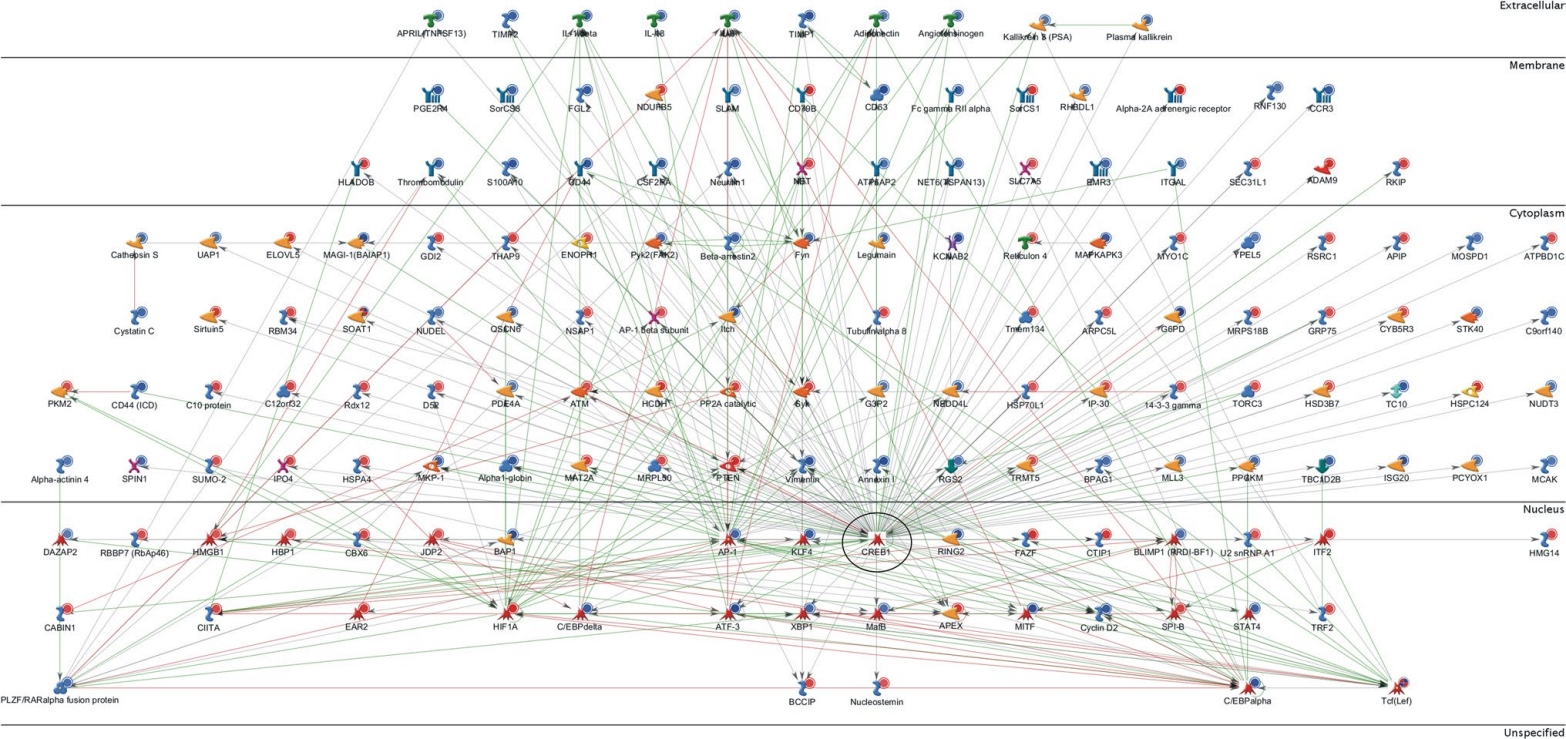
CREB Network. The CREB network shows the CREB transcription factor (enclosed by *black circle*) as a hub controlling the expression of proteins which are encoded by genes from the DE gene list. The various *symbols* used in the network have been described in detail in MetaCore Quick Reference Guide file publicly available at: https://portal.genego.com/help/MC_legend.pdf

**Fig. 5:**
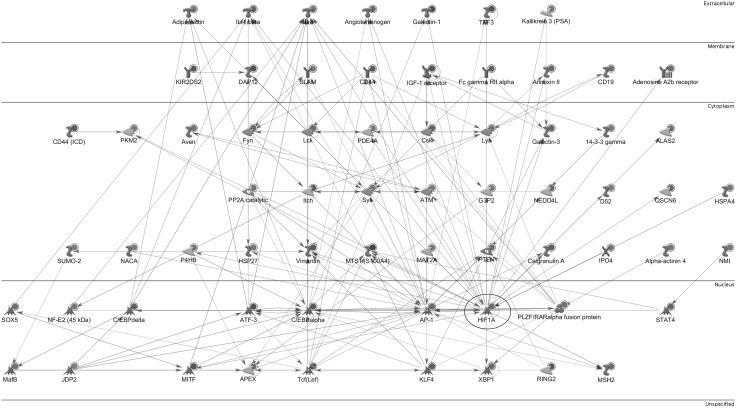
HIF1 Network. The HIF1 network shows the HIF1 transcription factor (enclosed by *black circle*) as a hub controlling the expression of proteins which are encoded by genes from the DE gene list. The various *symbols* used in the network have been described in detail in MetaCore Quick Reference Guide file publicly available at: https://portal.genego.com/help/MC_legend.pdf

We used the Analyzed Networks (AN) Transcription Regulation algorithm from MetaCore™ to identify the transcription factor (TF) pathways and networks that regulate the processes associated with BLV-induced pathogenesis. The AN Transcription Regulation algorithm revealed a list of 30 TFs potentially involved in the expression of differences observed in our study, including: CREB1, c-MYC, SP1, NF-κB, GCR-alpha, c-JUN, ETS1, C/EBP, p53, STAT1, STAT3 and HIF1A (supplementary MS Excel Table 9S). The three most connected factors: CREB1, c-MYC and SP1 regulate more than 50% of the genes in our DE data set. The CREB1 network (Fig. 4) illustrates CREB1 with its 159 targets (proteins regulated by CREB1) functioning in different areas of the cell. It should be emphasized that the BLV transactivator protein Tax binds and cooperates with the CREB transcription factor, and the former is one of the main targets of the Tax protein. Of note, transcription factors HIF1A (Fig. 5), C/EBP alpha, c-JUN (AP-1) which regulate the expression of many genes in our data, are simultaneously the targets for gene expression deregulation induced by BLV infection and the disease progression to the PL stage.

## Discussion

In this study we used oligonucleotide bovine specific BLOPlus microarrays and qRT-PCR analysis to examine gene expression changes in blood cells in response to retroviral BLV infection and disease progression to the lymphocytotic stage. Subsequently, the identified differentially expressed genes were used in the gene enrichment meta-analysis approach to reveal biological patterns associated with the expression differences observed. Before discussing the major items revealed by our study, we would like to emphasize the particular context of the obtained results. (1) The analysis of mRNA abundance between the naturally BLV-infected cattle and non-infected controls was performed on whole blood transcriptomes, using a dedicated tube system, which allowed for immediate cell lysis after bleeding, and the stabilization of received mRNAs with RNA stabilization solution. In contrast, most of the gene expression data, analyzed in the context of BLV studies and reported previously [22, 23, 50], were generated with peripheral blood mononuclear cells (PBMC) separated by density gradient centrifugation. As we also collected blood samples into tubes with EDTA, we endeavored to isolate total RNA also from buffy coats and PBMCs prepared with the standard Ficoll-Hypaque method. Probably due to the prolonged time required for blood collection and transport from dairy farms to the laboratory, exceeding 24 h, these methods resulted in partially degraded RNA with highly variable indices of RNA quality (RIN values ranging from 3.2 to 7.8, data not shown), which was not suitable for reliable gene expression analysis. In contrast, Tempus™ Blood RNA Tube technology allowed for RNA isolation with yields sufficient for microarray analysis and the RNA quality parameters indicating negligible RNA degradation between the groups in comparison (Table 1). In addition to RNA degradation, the reliability of gene expression estimations of many immunologically important genes (including those mentioned in the context of BLV response, like TNFα, IL6, IL10, IL12, IFNγ, NFκB, IκB) is significantly affected by unintended gene regulation caused by phlebotomy [35], sample handling, [47], and uncontrolled activation of coagulation [39]. Furthermore, in vivo BLV is latent in the majority of infected cells, however, expression of BLV in samples of whole blood from BLV-infected animals is activated immediately upon incubation at 37 °C in the absence of any exogenous factors, except for anticoagulants or the removal the blood cells from plasma [13, 46]. Consequently, an exaggerated immune response, based on differential mechanisms, could happen ex vivo in comparison to in vivo processes. Preservation of the blood transcriptomes in Tempus Blood RNA tubes or similar PAXgene tubes restricts ex vivo gene expression, allowing meaningful RNA assays and yielding transcript concentrations that are much closer to in vivo responses than can be obtained by other methods [39]. Nevertheless, the drawback of the whole blood transcriptome preservation approach is the fact that it is not possible at this time to distinguish which genes are deregulated because of their primal, direct participation in the host-pathogen interactions and which genes are affected as the result of secondary effects of BLV infection, such as the hematological profile alteration. Further investigations of selected genes reported in this study and involved in particular biological process of interest are needed to determine which molecular pathways result in BLV infection and disease progression and which are a result of BLV infection and disease progression. (2) The absence of clinically diagnosed lymphoma individuals that have been monitored in Poland in recent years and the comparatively high prevalence of animals with persistent lymphocytosis implied that a benign nonmalignant PL could be a convenient disease stage for the microarray analysis of host genetic factors that affect the outcome of BLV-infection. Lately, a strict BLV eradication policy has been implemented in Poland, and has resulted in the almost complete eradication of this previously highly endemic infectious agent. Newly diagnosed BLV seropositive individuals are eliminated from the herds immediately, and sometimes all animals within the herd (both seropositive and seronegative) are subjected to the compulsory slaughter. That is why we were able to collect blood samples from the BLV-positive individuals only once, so we could not examine the persistency of hematological aberrations and officially categorize them as the persistent lymphocytosis stage. However, the high total lymphocyte counts (mean 15.16 ± 10.28 × 10^3^/μl, Table 1), high total WBC counts, high titers of antibodies against p24 and gp51 and qualitative estimates of the proviral load (data not shown) [1, 16, 29] characterized all the animals within the BLV-positive group and indirectly indicated that all of them were PL.

The global gene expression data related to BLV-induced pathogenesis are relatively limited. To our knowledge, four research groups employed microarray technology to address potential gene expression alterations in host cells induced by BLV [4, 12, 22, 50]. In general, the lists of differentially expressed genes reported in these studies coincide sparsely with each other. A similar trend is also apparent when we compare the DE genes identified in this study with the previous reports. There are only a few DE genes in common between the lists (e.g. MHC class II-DOB, HAT1 encoding histone acetyltransferase 1, APEX1 encoding multifunctional DNA repair enzyme, CCNG1 encoding cyclin G1), a few DE genes with invert expression (e.g. JUN oncogene, MAFB encoding v-maf musculoaponeurotic fibrosarcoma oncogene homolog B), and a number of differentially expressed genes encoding members of closely related gene families with overlapping molecular functions (e.g. ATF-activating transcription factor family of basic leucine zipper protein members, LMO-LIM domain only family members of transcription regulators, DUSP-dual specificity phosphatase family members). The relatively small coincidence between the lists of DE genes among the analyzed reports should not be surprising, taking into account that different experimental designs were applied in each case. In three studies, transformed and immortalized cell lines were used, including human epithelial HeLa cells [4] and ovine jejunal Peyer’s patch-derived B-cell clone [22], which were subsequently transfected with plasmid vectors containing Tax_BLV,_ in order to identify the spectrum of host genes regulated by Tax. In the latter, PBMC derived sheep B-cells cultured in vitro were also analyzed. In the third study [12] and its follow-up with siRNA knock-down of BLV Tax [34], the transcript profiling of a transformed but uninfected bovine lymphoblastoid cell line BL3^o^ was performed in comparison to its in vitro BLV-infected derivative cell line BL3*. However, no details about the identity of DE genes were presented in either report. On the other hand, the genes up-regulated in PBMC from 3 PL cows, cultured with purified plasma blocking factor, as compared with those cultured in medium were shown [50]. Furthermore, different microarray platforms were applied, including singlE−color human specific Affymetrix chips [4], two-color human specific cross species microarray approaches [22, 50], and the two-color bovine specific 7 K cDNA microarray [12]. In contrast, this study presents a comparison of the blood transcriptomes between naturally BLV-infected cattle and uninfected controls, captured immediately after bleeding without cell culturing and performed with the bovine specific microarray platform.

In our data we noted the interesting phenomenon that BLV infection and disease progression to PL is associated with higher numbers of down-regulated genes than up-regulated ones (58% with FC ≥1.5 increasing to 65% when only FC ≥1.75 were considered). We hypothesize that, this may reflect the viral latency state, typical for the majority of BLV-infected cells in vivo and/or it may characterize BLV-induced immunosuppression, leading to the failure of an efficient immune response and underlying the host’s evasion mechanism developed by BLV during evolution. The opposite result, with the dominance of up-regulated genes (exceeding 70–80% of total DE genes), was reported in in vitro cultured cells induced by the BLV Tax protein [4, 22]. Such a redeployment towards gene induction is typical for many transcriptional profiles drawn from experiments utilizing in vitro cultures and purified factors in the study, dosed frequently with non-physiological ranges [9, 19, 50]. On the other hand, the results of the transcriptome profiling of blood cells from cattle naturally infected with pathogens like *Mycobacterium bovis, Mycobacterium avium* ssp. *paratuberculosis*, or *Trypanosoma congolense*, associated with chronic diseases, clearly demonstrated the importance of the gene repression mechanisms during the pathogenesis in vivo, which was reflected by the higher number of the down-regulated genes [21, 31, 33, 51]. The range of gene expression changes identified in our data set, with a maximum down- and up-regulation fold change factor of around 3.2, indicated that BLV-induced progression to the PL stage of the disease was associated mainly with low (FC ~1.5) and moderate (FC ≥1.75) gene expression changes, with only rare examples of high levels of differential expression (FC ≥3). A similar picture of primarily small and moderate gene expression changes emerged from the analysis of ovine B-cells transfected with the Tax containing vector [22]. Likewise, among 122 genes deregulated by the wild-type Tax protein only 10 showed differential expression exceeding fourfold, with the rest of the gene expression changes within the range of 2–3 fold [4]. Apart from genes with high levels of differential expression, those exhibiting low differences could be of peculiar concern, taking into account that small changes in gene expression are supposed to be adequate to disturb cell homeostasis [22].

Our expression data support the importance of DNA repair processes during BLV-induced leukemogenesis. Philpott and Buehring reported that in the immortalized and transformed cell lines infected by the HTLV/BLV group of retroviruses [38], Tax inhibits basE−excision DNA repair of oxidative damage, thereby potentially inducing genomic instability and increasing the accumulation of mutations in cellular DNA. Of note, we found an increased expression of APEX1 (also known asAPE1/Ref1) mRNA in the BLV-infected group (+2.05-fold, p = 1.58E−07). APEX1 encodes a multifunctional protein engaged in the DNA base excision repair (BER) pathway of oxidative DNA damage (apurinic/apyrimidinic-endonuclease activity), as well as in transcriptional regulation. The latter is achieved as a redox coactivator of many transcription factors (among others: AP-1, Egr-1, NF-κB, p53, and HIF), and by its ability to indirectly bind to the negative calcium response elements (nCaRE) of some promoters acting as a transcriptional repressor [2, 48]. The up-regulation of APEX1 mRNA was also reported in sheep B lymphocytes transfected with Tax-containing vector [22], and in tumor tissues and cancer cells of diverse origin [48], and its overexpression is associated with tumor cells’ resistance to various anticancer drugs [44]. The necessity of APEX1 for cellular survival and its frequent overexpression in tumor cells strongly suggest a fundamental role of this protein in preventing cell death and in controlling cellular proliferation [48], probably through its regulatory role in the expression of cyclin-dependent kinase inhibitor p21 (CDKN1A) [44] and in the DNA binding activity of the p53 protein [7]. Furthermore, we found MSH2 (MutS homolog 2) encoding an important component of the post-replicative DNA mismatch repair system (MMR) as the most significantly upregulated gene in the BLV-infected group (+2.25-fold, p = 7.54E−09, Table 3). MutSα may also play a role in DNA homologous recombination repair, and may modulate cell cycle regulation and apoptosis. Defects of MSH2 are implicated in the development of lymphoblastic lymphomas in humans and mice, and are associated with the aberrant expression of the LMO2 (LIM domain only 2) gene [28], encoding a transcription factor which has a central and crucial role in hematopoietic development and neoplastic transformation. It is of interest that we also observed an increase in the expression of LMO2 gene (+2.16-fold, p = 4.43E−07) in BLV-infected animals in comparison to the BLV-negative control group. In vitro studies indicated that genetic instability associated with the downregulating of MutSα alpha is induced by the HIF-1A transcription factor (Hypoxia inducible factor-1α) [24]. Hypoxia-oxygen deficiency in tissues is a key factor in the tumor microenvironment that has been tightly associated with tumor progression, metastasis and resistance to therapy [24] due to the increased rate of mutations in hypoxic conditions. We detected a significant over-expression of HIF-1A (+2.56-fold, p = 1.58E−07) in the BLV-positive group and our meta-analysis identifies an HIF1A network (Fig. 5) with the HIF1A protein as a hub with multiple biochemical connections leading potentially to EBL associated pathogenesis. The activation and involvement of hypoxia-inducible factor 1 in human T-cell leukemia virus type 1 (HTLV-1) infected cell lines and primary adult T-cell leukemia (ATLL) cells was reported previously by [49]. It is of particular interest because HTLV-1 and BLV are characterized by similar genomic organization, similar strategies for gene expression, and similar pathologies. Figure 5 also shows direct and mutual protein interactions between HIF1A and AP1 (cJUN) and C/EBPα. It could be of interest that both CEBPA and c-JUN genes were downregulated in BLV infected cows in comparison to control group (−2.32-fold, p = 9.3E−06 and −1.85-fold, p = 5.53E−03, respectively), increasing the effect of hypoxic factors [25, 43, 53] in BLV-associated disease progression.

In addition, the results of our transcription profiling indicted some aspects of innate immunity which could be impaired in BLV-infected animals. We noticed the differences in the mRNA expression levels of genes involved in the activation of the complement system, NK-cell cytotixicity and the TREM-1 signaling pathway. Namely, in the BLV-infected group we identified a decrease in the level of mRNAs for C1qA and C1qC constituents of the complement subcomponent C1q (−2.37-fold, p = 3.41E−03 and −1.9, p = 1.49E−03, respectively). This is of particular interest, because it has been reported previously that, the interaction and binding of human complement subcomponent C1q with the gp-21 protein of HTLV-1 inhibits its infectivity [17]. Moreover, we also found in BLV-infected cattle an increased level of C1qBP (C1q-binding protein) mRNA (+1.99-fold, p = 3.03E−06), encoding a multifunctional protein known to inhibit complement component C1 activation by binding to globular heads of C1q molecules. C1qBP is also involved in cell proliferation, migration and apoptosis, and has been reported to be upregulated in many human cancers [30]. Finally, we observed a decreased expression of CFD (adipsin, complement factor D), CFB (complement factor B) and CFP (properdin, complement factor P) genes (−3.21-fold, p = 1.74E−10; −1.93-fold, p = 4.25E−03 and −1.66-fold, p = 3.8E−03, respectively), which were involved in the alternative pathway of the complement system activation. The role of the alternative pathway in the response against retroviral agents is unclear, but some clues indicate their contribution to leukemogenesis. The CFD gene encodes a serine protease, which cleaves the complement factor B, releasing a small fragment Ba and a larger fragment Bb. The Ba fragment of complement factor B inhibits human B-lymphocyte proliferation [3], while the Bb fragment acts as B-cell growth factor [36] and is further complexed with C3b and properdin to form C5 convertase, participating in the complement cascade. Both adipsin and properdin genes were reported among 50 genes useful for leukemia class prediction, allowing the distinguishing of acute lymphoblastic leukemia (ALL) from acute myeloid leukemia (AML) patients [15].

Gene ennrichment analysis (GeneGo by process networks) identified the Inflammation NK-cell cytotoxicity network (Table 8S) as the most significantly affected category in our data set (FDR adjusted p value = 2.02-E03). This network comprises of 164 genes in total, and 18 of them showed differential expression in our microarray results. This is of interest as Natural Killer (NK) cells are lymphocytes of innate immunity that can participate in the lysis of some cancer cells and virus-infected cells with no previous activation. NK cells are also considered as important regulators of antiviral immune responses. In HTLV-1-infected individuals, the number of the NK cell was decreased and their activity was significantly impaired as compared to healthy controls [42]. Correspondingly, in BLV-infected cattle we found lower levels for mRNAs encoding NK-cell surface receptors, including KIR3DL3 (−2, threefold, p = 1.0E−04), NKR2B4 (CD244) (−1.56-fold, p = 1.48E−03), KLRC1 (NKG2A) (−1.77-fold, p = 4.77E−03) and NK-cell intracellular signaling proteins, including tyrosine kinases PTK2B (FAK2) (−1.77-fold, p = 1.55E−04),and its paralog SYK (+1.75-fold, p = 2.13E−05) and VAV3, encoding a guanine nucleotide exchange factor (−1.5-fold, p = 1.25E−03). In addition, a decreased level of TYROBP (DAP12) mRNA was observed (−2.54-fold, p = 1.83E−05). The TYROBP (TYRO Protein Tyrosine Kinase Binding Protein) gene encodes a transmembrane signaling protein, bearing an immunoreceptor tyrosinE−based activation motif (ITAM), and may be associated with the crosslinking of natural killer-cell inhibitory receptor (KIR) family of membrane glycoproteins and signal transduction leading to NK-cells activation. Besides its involvement in NK-cell cytotoxicity, TYROBP also plays a crucial role in the TREM-1 signaling pathway, which has recently been suggested as a novel mechanism in antiviral immunity [41]. Of interest is that TREM-1 signaling was also identified by enrichment analysis as the network significantly affected in our data set (Fig. 3b; Table 5S). Among genes with deregulated expression participating in the network we found TYROBP, SYK, protein kinase C inhibitor belonging to family 14-3-3 (YWHAG) (+1.57-fold, p = 2.7E−04), inositol 1,4,5-triphosphate receptor (ITPR1) (+1.57, p = 5.15E−03), caspase recruitment domain containing adaptor protein member 9 (CARD9) (−1.57-fold, p = 5.24E−03), tissue inhibitor of metalloproteinases 1 (TIMP1) (−2.4-fold, p = 6.30E−05), interleukin 8 (−1.58-fold p = 6.46E−03), and transcription factors c-JUN (−1.85-fold, p = 5.33E−03) and HMGB1 (+1.51, p = 0.16E−03). It seemed that BLV infection also affected the host’s innate immunity by the deregulation of the ubiquitin pathways and the proteasome degradation processes. In the BLV-infected group we observed a decreased level of mRNAs for ITCH, encoding itchy E3 ubiquitin protein ligase (−2.3-fold, p = 1.72E−07). Of interest, it was reported that, ITCH overexpression strongly inhibited the release and infectivity of wild-type HTLV-1 [10].

The differences in leukocyte cell subpopulations found among the BLV-infected and non-infected cattle groups may be partially responsible for some of the identified gene expression changes. Nevertheless, the data showed in this report also reinforce the hypothesis that a host- or pathogen- or both-driven mechanism of innate immune gene repression might be of crucial importance for the outcome of BLV infection under natural conditions. It could be of interest that the cattle in the non-infected control group but exposed to BLV for at least 3 years in farm conditions were marked with the increased levels of mRNA transcripts associated with innate immunity. Disruption or deficiency of a suitable innate immune response may thus be crucial in BLV dissemination and progression to disease. Due to the paucity of information on innate antiviral response to BLV infection and disease progression, further studies are clearly needed.

## Conclusion

Compared to earlier studies, we identified a significant number of new genes that have altered gene expression in BLV-infected blood cells. A higher number of down-regulated genes than up-regulated ones that have been noticed may reflect the viral latency state, typical for the majority of BLV-infected cells in vivo and/or it may characterize BLV-induced immunosupression. The latter could lead to the failure of efficient immune response and underlie the host evasion mechanism developed by BLV during evolution. Taking into account their function, the differentially expressed genes like: MSH2, APEX1, HIF1A, LMO2, CEBPA, and ITCH cast a new light on the mechanism of leukemogenesis associated with BLV and should be of particular interest in further studies. Furthermore, we have identified several new response pathways important for BLV-induced pathogenesis involving innate immunity including for the first time the complement system and TREM-1 signaling pathway, that are deregulated in BLV-infected cells. It provides a key for better understanding of the role of innate immunity against retroviral agents and gives a promise for successful intervening against them. However it should be emphasized that there are numerous regulatory processes that are targeted by BLV-infection and the complex network of interrelated pathways is instead disturbed, causing the interruption of the control of B-cell proliferation and programmed cell death.

## Electronic supplementary material

Below is the link to the electronic supplementary material.

**Figure 6S** Hierarchical clustering HCL heatmap for 370 differentially expressed genes (TIF 11385 KB)

**Table 2S** The complete list of 212 down-regulated genes in BLV-infected cattle in comparison to BLV-negative group according to decreasing value of B-statistics. (DOC 436 KB)

**Table 3S** The complete list of 158 up-regulated genes in BLV-infected cattle in comparison to BLV-negative group according to decreasing value of B-statistics. (DOC 235 KB)

**Table 5S** Enrichment analysis report by GO processes. (XLS 166 KB)

**Table 6S** Enrichment analysis report by GO molecular functions. (XLS 77 KB)

**Table 7S** Enrichment analysis report by GO localization. (XLS 110 KB)

**Table 8S** Enrichment analysis report by GeneGo process networks. (XLS 47 KB)

**Table 9S** Enrichment analysis report of transcription factors (TFs) involved with regulation of DE genes by Analyzed Networks (AN) transcription regulation analysis. (XLS 33 KB)
