# Supplementary material for: Microarray analysis of differential gene expression profiles in blood cells of naturally BLV-infected and uninfected Holstein–Friesian cows
Source: Mol Biol Rep. 2016 Nov 3;44(1):109–27. doi: 10.1007/s11033-016-4088-6 (PMC5310575; doi:10.1007/s11033-016-4088-6)
Supplement: Supplementary file 2 — Table 2S The complete list of 212 down-regulated genes in BLV-infected cattle in comparison to BLV-negative group according to decreasing value of B-statistics. (DOC 436 KB) [file 11033_2016_4088_MOESM2_ESM.doc]

Table 2S

The complete list of 212 down-regulated genes in BLV-infected cattle in comparison to BLV-negative group according to decreasing value of B-statistics.

| oligo_id | gene symbol | gene | Ref seq | Fold Change | p-value FDR adj. | B stat. |
| --- | --- | --- | --- | --- | --- | --- |
|  |  |  |  |  |  |  |
| Bt00001447 | CFD | complement factor D (adipsin) | NM_001034255 | -3,21 | 1,743E-10 | 22,5 |
| Bt00007539 | ITCH | itchy E3 ubiquitin protein ligase homolog (mouse) | NM_001082428 | -2,30 | 1,722E-07 | 14,1 |
| Bt00007239 | CD63 | CD63 molecule | NM_205803 | -2,57 | 1,722E-07 | 13,9 |
| Bt00000825 | AIF1 | allograft inflammatory factor 1 | NM_173985 | -3,13 | 2,151E-07 | 13,6 |
| BLO_ext_00163 | F5 | coagulation factor V (proaccelerin, labile factor) | NM_173879 | -2,50 | 2,744E-07 | 13,2 |
| Bt00005024 | S100A4 | S100 calcium binding protein A4 | NM_174595 | -3,21 | 3,990E-07 | 12,8 |
| Bt00002294 | LGALS1 | lectin, galactoside-binding, soluble, 1 | NM_175782 | -2,79 | 7,355E-07 | 12,0 |
| Bt00005520 | SLC40A1 | solute carrier family 40 (iron-regulated transporter), member 1 | NM_001077970 | -2,24 | 1,479E-06 | 11,2 |
| BLO_ext_00934 | SIRPA | signal-regulatory protein alpha | NM_175788 | -2,78 | 1,921E-06 | 10,9 |
| Bt00002021 | CST3 | cystatin C | NM_174029 | -1,83 | 2,586E-06 | 10,5 |
| BLO_ext_00656 | LGMN | legumain | NM_174101 | -2,02 | 5,908E-06 | 9,5 |
| Bt00004059 | NFAM1 | NFAT activating protein with ITAM motif 1 | XM_002687969 | -2,24 | 8,704E-06 | 9,1 |
| BLO_ext_01819 | CEBPA | CCAAT/enhancer binding protein (C/EBP), alpha | NM_176784 | -2,32 | 9,307E-06 | 9,0 |
| Bt00007592 | PLA2G7 | phospholipase A2, group VII (platelet-activating factor acetylhydrolase, plasma) | NM_174578 | -2,28 | 9,668E-06 | 8,9 |
| BLO_ext_00486 | CD2 | CD2 molecule | NM_001011676 | -2,21 | 9,766E-06 | 8,8 |
| Bt00003322 | STAT4 | signal transducer and activator of transcription 4 | NM_001083692 | -1,95 | 9,766E-06 | 8,8 |
| Bt00002910 | TGFBI | transforming growth factor, beta-induced, 68kDa | NM_001205402 | -2,71 | 1,164E-05 | 8,6 |
| Bt00007971 | AMICA1 | adhesion molecule, interacts with CXADR antigen 1 | NM_001080250 | -2,13 | 1,243E-05 | 8,5 |
| Bt00001417 | PLA2G16 | phospholipase A2, group XVI | NM_001075280 | -1,89 | 1,530E-05 | 8,3 |
| Bt00003194 | MTMR9 | myotubularin related protein 9-like | NM_001046256 | -2,26 | 1,673E-05 | 8,1 |
| BLO_ext_00190 | TYROBP | TYRO protein tyrosine kinase binding protein | NM_174627 | -2,54 | 1,833E-05 | 8,0 |
| Bt00007151 | ANXA3 | annexin A3 | NM_001035325 | -1,81 | 1,872E-05 | 7,9 |
| Bt00007329 | LGALS3 | lectin, galactoside-binding, soluble, 3 | NM_001102341 | -1,89 | 2,056E-05 | 7,8 |
| BLO_ext_01078 | CAPN2 | calpain 2, (m/II) large subunit | NM_001103086 | -1,83 | 2,220E-05 | 7,7 |
| Bt00003792 | ITGB7 | integrin, beta 7 | NM_001105365 | -1,93 | 2,363E-05 | 7,6 |
| Bt00007720 | IPCEF1 | interaction protein for cytohesin exchange factor 1 | XM_595033 | -1,62 | 2,471E-05 | 7,5 |
| Bt00006837 | CD9 | CD9 molecule | NM_173900 | -1,73 | 2,471E-05 | 7,5 |
| Bt00007984 | MITF | microphthalmia-associated transcription factor | NM_001001150 | -2,47 | 2,731E-05 | 7,4 |
| Bt00000816 | S100A10 | S100 calcium binding protein A10 | NM_174650 | -2,32 | 2,785E-05 | 7,4 |
| Bt00002561 | DUSP6 | dual specificity phosphatase 6 | NM_001046195 | -1,95 | 2,822E-05 | 7,3 |
| Bt00006763 | CTSC | cathepsin C | NM_001033617 | -1,91 | 4,754E-05 | 6,7 |
| Bt00001685 | CD44 | CD44 molecule | NM_174013 | -2,17 | 5,555E-05 | 6,5 |
| Bt00001603 | PTGER4 | prostaglandin E receptor 4 | NM_174589 | -2,26 | 5,664E-05 | 6,5 |
| Bt00007096 | KIAA1598 | KIAA1598 ortholog | XR_139692 | -2,14 | 6,304E-05 | 6,4 |
| BLO_ext_01426 | TIMP1 | TIMP metallopeptidase inhibitor 1 | NM_174471 | -2,40 | 6,304E-05 | 6,3 |
| Bt00003234 | IGHA1 | Bos taurus IgM | NM_001205186 | -2,48 | 6,906E-05 | 6,2 |
| Bt00004679 | VIM | vimentin | NM_173969 | -2,35 | 7,332E-05 | 6,1 |
| Bt00001158 | SULT1A1 | sulfotransferase family, cytosolic, 1A, phenol-preferring, member | NM_177521 | -2,20 | 7,497E-05 | 6,1 |
| BLO_ext_00611 | IL17R | nterleukin 17 receptor | XM_005895201 | -1,75 | 9,065E-05 | 5,9 |
| Bt00002286 | FCN2 | ficolin (collagen/fibrinogen domain containing lectin) 2 (hucolin) | NM_001010996 | -2,48 | 9,543E-05 | 5,8 |
| BLO_ext_00855 | WIPF1 | AS/WASL interacting protein family, member 1 | NM_001076923 | -1,73 | 9,543E-05 | 5,8 |
| Bt00003812 | S100A8 | S100 calcium binding protein A8 (S100A8) | NM_001113725 | -2,07 | 9,925E-05 | 5,7 |
| BLO_ext_01638 | KIR3DL3 | killer cell immunoglobulin-like receptor, three domains, long cytoplasmic tail, 3 | XR_138959 | -2,30 | 1,000E-04 | 5,7 |
| Bt00001056 | USP11 | ubiquitin specific peptidase 11 | NM_001080308 | -2,03 | 1,040E-04 | 5,6 |
| BLO_ext_00351 | TXK | TXK tyrosine kinase | NM_001206148 | -1,70 | 1,070E-04 | 5,6 |
| Bt00005014 | HRSP12 | heat-responsive protein 12 | NM_001034208 | -1,75 | 1,096E-04 | 5,5 |
| BLO_ext_00649 | COTL1 | coactosin-like 1 (Dictyostelium) | NM_001046593 | -1,66 | 1,186E-04 | 5,4 |
| Bt00003133 | SMIM10 | small integral membrane protein 10 | NM_001163442 | -1,81 | 1,311E-04 | 5,3 |
| Bt00003869 | RHBDL1 | rhomboid, veinlet-like 1 (Drosophila) | XM_002697968 | -1,60 | 1,358E-04 | 5,3 |
| BLO_ext_01145 | THBD | thrombomodulin | NM_001166522 | -1,90 | 1,417E-04 | 5,2 |
| Bt00005806 | PDE2A | phosphodiesterase 2A, cGMP-stimulated | NM_001143846 | -1,71 | 1,458E-04 | 5,2 |
| BLO_ext_00132 | ANG | angiogenin, ribonuclease, RNase A family, 5 | NM_001078144 | -1,80 | 1,436E-04 | 5,2 |
| Bt00000492 | QSOX1 | quiescin Q6 sulfhydryl oxidase 1 | NM_001102074 | -1,93 | 1,528E-04 | 5,1 |
| BLO_ext_01196 | IL1B | interleukin 1, beta | NM_174093 | -2,00 | 1,551E-04 | 5,1 |
| BLO_ext_00595 | PTK2B | PTK2B protein tyrosine kinase 2 beta | NM_001102252 | -1,77 | 1,551E-04 | 5,1 |
| BLO_ext_00585 | FCGR3A | Fc fragment of IgG, low affinity IIIa, receptor (CD16a)(FCGR3A) | NM_001077402 | -2,00 | 1,601E-04 | 5,1 |
| Bt00006721 | FCHO2 | FCH domain only 2 | NM_001098119 | -1,74 | 1,604E-04 | 5,0 |
| Bt00007625 | PGRMC1 | progesterone receptor membrane component 1 | NM_001075133 | -1,91 | 1,827E-04 | 4,9 |
| Bt00007691 | SORL1 | sortilin-related receptor, L(DLR class) A repeats containing | NM_001192757 | -2,10 | 1,827E-04 | 4,9 |
| Bt00006930 | TKT | transketolase | NM_001003906 | -1,66 | 1,913E-04 | 4,8 |
| Bt00001653 | DYNLT1 | dynein, light chain, Tctex-type 1 | NM_174620 | -1,90 | 1,999E-04 | 4,7 |
| Bt00008215 | NDEL1 | nudE nuclear distribution gene E homolog (A.nidulans)-like 1 | NM_001191246 | -1,58 | 2,052E-04 | 4,7 |
| BLO_ext_00634 | FYN | FYN oncogene related to SRC | NM_001077972 | -1,86 | 2,073E-04 | 4,7 |
| Bt00007197 | ANXA1 | annexin A1 | NM_175784 | -2,54 | 2,335E-04 | 4,5 |
| Bt00003761 | PYCARD | PYD and CARD domain containing | NM_174730 | -1,63 | 2,403E-04 | 4,5 |
| Bt00000738 | LYZ2 | lysozyme C-2 | NM_180999 | -2,46 | 2,584E-04 | 4,4 |
| BLO_ext_01755 | STOM | stomatin | NM_001105473 | -1,95 | 2,584E-04 | 4,4 |
| Bt00004396 | MAPKAPK3 | mitogen-activated protein kinase-activated protein kinase 3 | NM_001034779 | -2,17 | 2,800E-04 | 4,3 |
| BLO_ext_00616 | SPSB2 | splA/ryanodine receptor domain and SOCS box containing 2 | NM_001076280 | -1,54 | 3,225E-04 | 4,1 |
| Bt00000320 | GLIPR2 | GLI pathogenesis-related 2 | NM_001076112 | -1,94 | 3,487E-04 | 4,0 |
| Bt00005715 | ATP6AP2 | ATPase, H+ transporting, lysosomal accessory protein 2 | NM_001098022 | -1,56 | 3,580E-04 | 4,0 |
| Bt00001768 | ATXN1 | ataxin 1 | XM_005223825 | -1,77 | 3,593E-04 | 4,0 |
| Bt00006839 | CSK | c-src tyrosine kinase | NM_001075397 | -2,03 | 3,593E-04 | 4,0 |
| BLO_ext_00620 | CXCR2 | interleukin 8 receptor, beta | NM_174360 | -2,51 | 3,948E-04 | 3,9 |
| Bt00003998 | RHOQ | ras homolog gene family, member Q | NM_001205498 | -2,21 | 3,986E-04 | 3,9 |
| Bt00002518 | C1orf21 | BTA 16 open reading frame, human C1orf21 | NM_001081547 | -2,08 | 4,195E-04 | 3,8 |
| Bt00008172 | ANXA2 | annexin A2 | NM_174716 | -1,81 | 4,195E-04 | 3,8 |
| Bt00005900 | CDKN2D | cyclin-dependent kinase inhibitor 2D (p19, inhibits CDK4) | NM_001046050 | -1,81 | 4,293E-04 | 3,8 |
| Bt00000880 | ITGA6 | integrin, alpha 6 | NM_001109981 | -1,93 | 5,026E-04 | 3,6 |
| Bt00003191 | RNASE6 | ribonuclease, RNase A family, k6 | NM_174594 | -1,76 | 5,512E-04 | 3,5 |
| Bt00008047 | PCK2 | phosphoenolpyruvate carboxykinase 2 | NM_001205594 | -1,76 | 5,729E-04 | 3,4 |
| Bt00007652 | CTSW | cathepsin W | NM_001110070 | -1,90 | 5,742E-04 | 3,4 |
| Bt00001126 | CD2BP2 | CD2 (cytoplasmic tail) binding protein 2 | NM_001083682 | -1,60 | 5,961E-04 | 3,4 |
| Bt00003606 | MACROD1 | MACRO domain containing 1 | NM_001046509 | -1,53 | 6,119E-04 | 3,3 |
| Bt00003755 | GNG5 | guanine nucleotide binding protein (G protein), gamma 5 | NM_174811 | -1,49 | 6,233E-04 | 3,3 |
| BLO_ext_01047 | CERKL | ceramide kinase-like | XM_003581805 | -1,97 | 6,311E-04 | 3,3 |
| Bt00006188 | P4HB | prolyl 4-hydroxylase, beta polypeptide | NM_174135 | -1,56 | 6,540E-04 | 3,2 |
| Bt00002015 | SELPLG | P-selectin glycoprotein ligand 1 mRNA | NM_001037628 | -1,74 | 6,677E-04 | 3,2 |
| Bt00001472 | LCK | lymphocyte-specific protein tyrosine kinase | NM_001034334 | -1,85 | 6,740E-04 | 3,2 |
| Bt00007046 | ADIPOQ | adiponectin, C1Q and collagen domain | NM_174742 | -1,52 | 6,740E-04 | 3,2 |
| Bt00003214 | SLC7A7 | solute carrier family 7 (amino acid transporter light chain, y+L system) member 7 | NM_001075151 | -1,73 | 6,760E-04 | 3,2 |
| Bt00007067 | GPT2 | glutamic pyruvate transaminase (alanine aminotransferase) | XM_005218738 | -1,79 | 6,873E-04 | 3,2 |
| Bt00003984 | MEF2BNB | MEF2B neighbor (MEF2BNB) | NM_001145792 | -1,90 | 6,880E-04 | 3,1 |
| Bt00005446 | LCP2 | lymphocyte cytosolic protein 2 (SH2 domain containing leukocyte protein of 76kDa) | NM_001076844 | -1,73 | 7,476E-04 | 3,1 |
| BLO_ext_00429 | KMT2C | lysine (K)-specific methyltransferase 2C | XM_005198184 | -1,55 | 7,567E-04 | 3,0 |
| Bt00001530 | DUSP1 | dual specificity phosphatase 1 | NM_001046452 | -1,90 | 8,163E-04 | 2,9 |
| Bt00006873 | IGF1R | insulin-like growth factor 1 receptor | NM_001244612 | -2,36 | 8,163E-04 | 2,9 |
| Bt00007076 | XBP1 | X-box binding protein 1 | NM_001034727 | -1,83 | 8,468E-04 | 2,9 |
| Bt00007796 | RABGAP1L | RAB GTPase activating protein 1-like | XM_003587088 | -1,94 | 8,448E-04 | 2,9 |
| Bt00006480 | LXN | latexin | NM_001080340 | -1,80 | 9,127E-04 | 2,8 |
| BLO_ext_01149 | ITGAL | integrin, alpha L (antigen CD11A (p180), lymphocyte function-associated antigen 1; alpha polypeptide) | XM_005224830 | -1,47 | 9,316E-04 | 2,8 |
| Bt00004490 | WLS | wntless homolog (Drosophila) | XM_005204461 | -2,33 | 9,445E-04 | 2,7 |
| Bt00007308 | SDCBP | syndecan binding protein (syntenin) | NM_001075483 | -1,52 | 1,003E-03 | 2,7 |
| BLO_ext_00241 | CD3E | CD3e molecule, epsilon (CD3-TCR complex) | NM_174011 | -2,18 | 1,004E-03 | 2,7 |
| Bt00005131 | CDK5R1 | cyclin-dependent kinase 5, regulatory subunit 1 (p35) | NM_174512 | -1,79 | 1,057E-03 | 2,6 |
| BLO_ext_01656 | A2M | alpha-2-macroglobulin | NM_001109795 | -1,74 | 1,080E-03 | 2,6 |
| Bt00008252 | TSPAN13 | tetraspanin 13 | NM_001035362 | -1,82 | 1,162E-03 | 2,5 |
| Bt00004937 | TBC1D2B | TBC1 domain family, member 2B | XM_002696662 | -2,01 | 1,168E-03 | 2,5 |
| BLO_ext_01157 | SELL | selectin L, CD62, LECAM1 | NM_174182 | -1,83 | 1,209E-03 | 2,4 |
| BLO_ext_01292 | CD3G | CD3g molecule, gamma (CD3-TCR complex) | NM_001040472 | -1,44 | 1,236E-03 | 2,4 |
| Bt00007185 | VAV3 | vav 3 guanine nucleotide exchange factor | XM_002686162 | -1,46 | 1,256E-03 | 2,4 |
| BLO_ext_01633 | KIF2C | kinesin family member 2C | NM_001101147 | -1,52 | 1,270E-03 | 2,4 |
| Bt00001258 | ARRB2 | arrestin, beta 2 | XM_005220181 | -1,98 | 1,341E-03 | 2,3 |
| Bt00004670 | EFHD2 | EF-hand domain family, member D2 | NM_001103245 | -1,52 | 1,377E-03 | 2,3 |
| Bt00007180 | RASGEF1B | RasGEF domain family, member 1B | NM_001083649 | -1,72 | 1,457E-03 | 2,2 |
| BLO_ext_00345 | CD244 | CD244 molecule, natural killer cell receptor 2B4 | NM_001192350 | -1,56 | 1,479E-03 | 2,2 |
| Bt00001250 | C1QC | complement component 1, q subcomponent, C chain (C1QC) | NM_001206396 | -1,90 | 1,496E-03 | 2,1 |
| Bt00002527 | RNF130 | ring finger protein 130 | NM_001099708 | -1,55 | 1,521E-03 | 2,1 |
| Bt00000211 | NEDD4L | neural precursor cell expressed, developmentally down-regulated 4-like, E3 ubiquitin protein ligase | XM_005199752 | -1,89 | 1,606E-03 | 2,1 |
| Bt00008084 | CYP27A1 | cytochrome P450, family 27, subfamily A, polypeptide 1 | NM_001083413 | -1,53 | 1,639E-03 | 2,0 |
| BLO_ext_01623 | CEBPD | CCAAT/enhancer binding protein (C/EBP), delta | NM_174267 | -2,09 | 1,659E-03 | 2,0 |
| Bt00008067 | UAP1 | UDP-N-acteylglucosamine pyrophosphorylase 1 | XM_005203527 | -1,58 | 1,736E-03 | 2,0 |
| Bt00007310 | CHI3L1 | chitinase 3-like 1 | NM_001080219 | -1,76 | 1,761E-03 | 1,9 |
| Bt00001168 | SOAT1 | sterol O-acyltransferase 1 | NM_001034206 | -1,48 | 1,842E-03 | 1,9 |
| Bt00003013 | KLF4 | Kruppel-like factor 4 | NM_001105385 | -2,12 | 1,858E-03 | 1,9 |
| Bt00004768 | CPD | carboxypeptidase D | XM_003587391 | -1,53 | 1,858E-03 | 1,9 |
| BLO_ext_01756 | NFE2 | nuclear factor (erythroid-derived 2), 45kDa | NM_001014923 | -1,47 | 1,858E-03 | 1,9 |
| Bt00006164 | HK3 | hexokinase 3 | NM_001101929 | -1,52 | 1,876E-03 | 1,9 |
| Bt00005849 | NRSN2 | neurensin 2 | NM_001101269 | -1,56 | 1,923E-03 | 1,8 |
| Bt00004882 | RARA | retinoic acid receptor, alpha | NM_001014942 | -1,67 | 1,984E-03 | 1,8 |
| Bt00004279 | MXD4 | MAX dimerization protein 4 | NM_001076256 | -1,61 | 2,046E-03 | 1,8 |
| Bt00002735 | CYB5R1 | cytochrome b5 reductase 1 | NM_001034518 | -2,02 | 2,124E-03 | 1,7 |
| Bt00007286 | TALDO1 | transaldolase 1 | NM_001035283 | -1,76 | 2,231E-03 | 1,7 |
| BLO_ext_01352 | FGL2 | fibrinogen-like 2 | NM_001046097 | -2,11 | 2,253E-03 | 1,6 |
| Bt00001499 | NRN1 | neuritin 1 | NM_001046438 | -2,19 | 2,253E-03 | 1,6 |
| BLO_ext_c0001 | GAPDH | Glyceraldehyde-3-phosphate dehydrogenase | NM_001034034 | -1,47 | 2,408E-03 | 1,6 |
| BLO_ext_00744 | ZMYM6 | zinc finger, MYM-type 6 | NM_001206292 | -1,53 | 2,426E-03 | 1,5 |
| Bt00002656 | DNAJC1 | DnaJ (Hsp40) homolog, subfamily C, member 1 | XM_003582915 | -1,44 | 2,508E-03 | 1,5 |
| BLO_ext_01165 | SLAMF1 | signaling lymphocytic activation molecule family member 1 | NM_174184 | -1,46 | 2,550E-03 | 1,5 |
| Bt00002129 | LRRC25 | leucine rich repeat containing 25 | NM_174688 | -1,84 | 2,614E-03 | 1,5 |
| Bt00001716 | STK40 | serine/threonine kinase 40 | NM_001075727 | -1,69 | 2,657E-03 | 1,4 |
| Bt00000856 | LAT | linker for activation of T cells | NM_001104978 | -1,82 | 2,676E-03 | 1,4 |
| Bt00006585 | CTSS | cathepsin S | NM_001033615 | -1,78 | 2,703E-03 | 1,4 |
| Bt00004483 | PPP1R3B | protein phosphatase 1, regulatory subunit 3B | NM_001103247 | -1,77 | 2,703E-03 | 1,4 |
| Bt00005239 | EMR3 | egf-like module containing, mucin-like, hormone receptor-like 3 | XM_005228632 | -1,83 | 2,714E-03 | 1,4 |
| Bt00001594 | RGS2 | regulator of G-protein signaling 2, 24kDa | NM_001075596 | -1,69 | 2,723E-03 | 1,4 |
| Bt00004006 | ALAS2 | aminolevulinate, delta-, synthase 2 | NM_001035103 | -1,79 | 2,748E-03 | 1,4 |
| BLO_ext_00885 | CSF2RA | granulocyte-macrophage colony-stimulating factor receptor subunit alpha-like | XM_005192580 | -2,07 | 2,781E-03 | 1,3 |
| Bt00001301 | GSN | gelsolin | NM_001113284 | -1,87 | 2,891E-03 | 1,3 |
| Bt00002662 | SLC46A2 | solute carrier family 46, member 2 | NM_001024519 | -1,84 | 2,903E-03 | 1,3 |
| Bt00001526 | MAFB | v-maf avian musculoaponeurotic fibrosarcoma oncogene homolog B | XM_610891 | -1,62 | 2,916E-03 | 1,3 |
| BLO_ext_00654 | KLKB1 | kallikrein B, plasma (Fletcher factor) 1 | NM_001046352 | -1,88 | 2,967E-03 | 1,3 |
| Bt00002062 | PLBD1 | phospholipase B domain containing 1 | NM_001166298 | -2,04 | 2,967E-03 | 1,3 |
| Bt00005322 | SORCS3 | sortilin-related VPS10 domain containing receptor 3 | XM_005225556 | -1,90 | 3,091E-03 | 1,2 |
| Bt00007416 | DAZAP2 | DAZ associated protein 2 | XM_005206320 | -1,45 | 3,152E-03 | 1,2 |
| Bt00006192 | LEF1 | lymphoid enhancer-binding factor 1 | XM_005207653 | -1,86 | 3,295E-03 | 1,1 |
| Bt00008085 | CCR3 | chemokine (C-C motif) receptor 3 | XM_005223027 | -2,15 | 3,338E-03 | 1,1 |
| BLO_ext_01685 | MEX3A | mex-3 RNA binding family member A | NM_001206290 | -1,47 | 3,338E-03 | 1,1 |
| Bt00007788 | C1QA | complement component 1, q subcomponent, A chain | NM_001014945 | -2,37 | 3,410E-03 | 1,1 |
| Bt00002057 | OSBPL7 | oxysterol binding protein-like 7 | NM_001205647 | -1,52 | 3,454E-03 | 1,1 |
| Bt00006064 | ATPIF1 | ATPase inhibitory factor 1 | NM_175816 | -2,33 | 3,478E-03 | 1,0 |
| BLO_ext_00863 | IL18 | interleukin 18 (interferon-gamma-inducing factor) | XM_005215801 | -1,85 | 3,478E-03 | 1,0 |
| Bt00002556 | NCF1 | neutrophil cytosolic factor 1 | NM_174119 | -2,01 | 3,495E-03 | 1,0 |
| Bt00000495 | MZB1 | marginal zone B and B1 cell-specific protein | NM_001098930 | -1,86 | 3,532E-03 | 1,0 |
| Bt00008066 | YPEL5 | yippee-like 5 (Drosophila) | NM_001079793 | -1,46 | 3,778E-03 | 0,9 |
| Bt00000454 | DST | dystonin | XM_001252266 | -1,71 | 3,801E-03 | 0,9 |
| Bt00006419 | PF4,CXCL4 | platelet factor 4 | NM_001101062 | -1,99 | 3,801E-03 | 0,9 |
| BLO_ext_01712 | CFP | complement factor properdin | NM_001076178 | -1,66 | 3,805E-03 | 0,9 |
| Bt00000808 | ACTN4 | actinin, alpha 4 | NM_001098052 | -1,47 | 3,815E-03 | 0,9 |
| Bt00006306 | CDC42EP3 | CDC42 effector protein (Rho GTPase binding) 3 | NM_001046444 | -2,11 | 3,946E-03 | 0,9 |
| Bt00004234 | TSPAN17 | tetraspanin 17 | NM_001014880 | -1,60 | 3,959E-03 | 0,9 |
| BLO_ext_00004 | NUDT3 | nudix (nucleoside diphosphate linked moiety X)-type motif 3 | NM_001082466 | -1,50 | 4,100E-03 | 0,8 |
| Bt00002956 | IL6R | interleukin 6 receptor | NM_001110785 | -1,77 | 4,133E-03 | 0,8 |
| BLO_ext_01417 | CFB | complement factor B | NM_001040526 | -1,93 | 4,252E-03 | 0,8 |
| Bt00007147 | LPAR6 | lysophosphatidic acid receptor 6 | NM_001101284 | -1,70 | 4,300E-03 | 0,8 |
| Bt00000290 | SLC31A2 | solute carrier family 31 (copper transporters) | NM_001034556 | -1,77 | 4,356E-03 | 0,7 |
| Bt00001322 | PHF12 | PHD finger protein 12 | NM_001192131 | -1,85 | 4,411E-03 | 0,7 |
| Bt00006746 | IGG1C | IgG1 heavy chain constant region (IgC-gamma) | S82409 | -1,82 | 4,547E-03 | 0,7 |
| Bt00007924 | HNMT | histamine N-methyltransferase | NM_001035434 | -1,67 | 4,736E-03 | 0,6 |
| BLO_ext_01363 | KLRC1 | killer cell lectin-like receptor subfamily C, member 1 | NM_001168587 | -1,77 | 4,777E-03 | 0,6 |
| Bt00001486 | IGJ | immunoglobulin J polypeptide, linker protein for immunoglobulin alpha and mu polypeptides | NM_175773 | -1,88 | 4,872E-03 | 0,6 |
| Bt00001995 | CA2 | carbonic anhydrase II | NM_178572 | -2,83 | 4,928E-03 | 0,6 |
| Bt00008258 | CD96 | CD96 molecule | NM_001035072 | -1,71 | 5,205E-03 | 0,5 |
| Bt00005134 | CARD9 | caspase recruitment domain family, member 9 | NM_001077111 | -1,57 | 5,244E-03 | 0,5 |
| Bt00004832 | BAP1 | BRCA1 associated protein-1 (ubiquitin carboxy-terminal hydrolase) | NM_001102549 | -2,50 | 5,244E-03 | 0,5 |
| Bt00001800 | PDE4A | phosphodiesterase 4A, cAMP-specific | NM_001101081 | -1,48 | 5,330E-03 | 0,5 |
| Bt00004743 | JUN | jun proto-oncogene | NM_001077827 | -1,85 | 5,331E-03 | 0,5 |
| Bt00003624 | NDUFA4 | NADH dehydrogenase (ubiquinone) 1 alpha subcomplex, 4 | NM_175820 | -1,54 | 5,465E-03 | 0,4 |
| Bt00002217 | PRDM1 | PR domain containing 1, with ZNF domain | NM_001192936 | -1,72 | 5,612E-03 | 0,4 |
| BLO_ext_01321 | LGALSL | lectin, galactoside-binding-like | NM_001205831 | -1,60 | 5,613E-03 | 0,4 |
| BLO_ext_01813 | DEFB7 | defensin beta 7 | NM_001102362 | -1,70 | 5,966E-03 | 0,3 |
| BLO_ext_01611 | TIMP2 | TIMP metallopeptidase inhibitor 2 | NM_174472 | -2,04 | 6,105E-03 | 0,3 |
| BLO_ext_00786 | CXCL8 | interleukin 8 | NM_173925 | -1,56 | 6,464E-03 | 0,2 |
| Bt00002477 | CC2D1B | coiled-coil and C2 domain containing 1B | XM_588670 | -1,53 | 6,779E-03 | 0,2 |
| BLO_ext_01171 | MKNK1 | MAP kinase interacting serine/threonine kinase 1 | NM_001035358 | -1,57 | 6,859E-03 | 0,2 |
| Bt00001520 | ISG20 | interferon stimulated exonuclease gene 20kDa | XM_005911016 | -2,44 | 6,934E-03 | 0,1 |
| Bt00005300 | HBA | hemoglobin, alpha 2 | NM_001077422 | -2,16 | 6,991E-03 | 0,1 |
| Bt00004637 | ARHGAP9 | Rho GTPase activating protein 9 | XM_005192327 | -1,49 | 6,991E-03 | 0,1 |
| BLO_ext_01233 | LAMP1 | lysosomal-associated membrane protein 1 | NM_001075124 | -1,53 | 7,158E-03 | 0,1 |
| Bt00006409 | SNX10 | sorting nexin 10 | NM_001075375 | -1,65 | 7,440E-03 | 0,1 |
| Bt00007336 | DSTN | destrin (actin depolymerizing factor) | XM_005904567 | -1,62 | 7,791E-03 | 0,0 |
| Bt00002295 | PARP8 | poly (ADP-ribose) polymerase family, member 8 | NM_001192298 | -1,53 | 7,925E-03 | 0,0 |
| Bt00000864 | G6PD | glucose-6-phosphate dehydrogenase | NM_001244135 | -3,07 | 7,952E-03 | 0,0 |
| Bt00001709 | CD84 | CD84 molecule | XM_002685862 | -1,55 | 8,315E-03 | -0,1 |
| BLO_ext_00800 | HSPB1 | heat shock 27kDa protein 1 | NM_001025569 | -1,53 | 8,638E-03 | -0,1 |
| Bt00003441 | MOSPD1 | motile sperm domain containing 1 | NM_001038137 | -1,56 | 8,667E-03 | -0,1 |
| Bt00005762 | ATF3 | activating transcription factor 3 | NM_001046193 | -2,64 | 8,804E-03 | -0,1 |
| Bt00000626 | TNFSF13 | tumor necrosis factor (ligand) superfamily, member 13 | NM_001034647 | -1,60 | 8,918E-03 | -0,2 |
| Bt00006632 | PCYOX1 | prenylcysteine oxidase 1 | NM_001105474 | -1,45 | 8,969E-03 | -0,2 |
| Bt00007240 | SLC44A1 | solute carrier family 44 (choline transporter), member 1 | XM_002689908 | -1,57 | 9,037E-03 | -0,2 |
| Bt00005786 | FKBP11 | FK506 binding protein 11, 19 kDa | NM_001045932 | -1,45 | 9,336E-03 | -0,2 |
| Bt00004349 | GUCY1B3 | guanylate cyclase 1, soluble, beta 3 | NM_174641 | -2,14 | 9,854E-03 | -0,3 |
|  |  |  |  |  |  |  |
