# Supplementary material for: Microarray analysis of differential gene expression profiles in blood cells of naturally BLV-infected and uninfected Holstein–Friesian cows
Source: Mol Biol Rep. 2016 Nov 3;44(1):109–27. doi: 10.1007/s11033-016-4088-6 (PMC5310575; doi:10.1007/s11033-016-4088-6)
Supplement: Supplementary file 3 — Table 3S The complete list of 158 up-regulated genes in BLV-infected cattle in comparison to BLV-negative group according to decreasing value of B-statistics. (DOC 235 KB) [file 11033_2016_4088_MOESM3_ESM.doc]

Table 3S

The complete list of 158 up-regulated genes in BLV-infected cattle in comparison to BLV-negative group according to decreasing value of B-statistics.

| oligo_id | gene symbol | gene | Ref seq | fold change | p-value FDR adj. | B stat. |
| --- | --- | --- | --- | --- | --- | --- |
|  |  |  |  |  |  |  |
| Bt00004943 | MSH2 | mutS homolog 2, colon cancer, nonpolyposis type 1 (E. coli) | NM_001034584 | 2,25 | 7,54E-09 | 18,5 |
| Bt00004141 | KBTBD8 | T cell activation kelch repeat protein | NM_001192696 | 2,66 | 1,78E-08 | 17,3 |
| Bt00006375 | ADRA2A | alpha2A adrenergic receptor | NM_174499 | 3,18 | 2,39E-08 | 16,8 |
| BLO_ext_00185 | MS4A1, CD20 | membrane-spanning 4-domains, subfamily A, member 1 | NM_001077854 | 2,95 | 2,39E-08 | 16,6 |
| BLO_ext_00550 | ADORA2B | adenosine A2b receptor | NM_001075925 | 2,38 | 8,08E-08 | 15,3 |
| Bt00002242 | PPA2 | pyrophosphatase (inorganic) 2 | NM_001076396 | 2,16 | 1,34E-07 | 14,7 |
| Bt00000295 | CD19 | CD19 molecule | NM_001245998 | 2,12 | 1,58E-07 | 14,3 |
| BLO_ext_00374 | APEX1 | APEX nuclease (multifunctional DNA repair enzyme) 1 | NM_176609 | 2,05 | 1,58E-07 | 14,3 |
| Bt00000725 | HIF1A | hypoxia inducible factor 1, alpha subunit (basic helix-loop-helix transcription factor) | NM_174339 | 2,56 | 1,72E-07 | 14,0 |
| BLO_ext_01490 | SLC4A10 | Solute carrier family 4, sodium bicarbonate  transporter-like, member 10 | NM_001038128 | 3,05 | 2,27E-07 | 13,5 |
| Bt00006625 | CCT5 | T-complex protein 1, chaperonin containing TCP1, subunit 5 (epsilon) | NM_001034595 | 1,75 | 2,48E-07 | 13,4 |
| Bt00005595 | SH3GLB2 | SH3-domain GRB2-like endophilin B2 | NM_001076802 | 2,13 | 4,43E-07 | 12,6 |
| Bt00001908 | LMO2 | LIM domain only 2 (rhombotin-like 1) | NM_001076352 | 2,16 | 4,43E-07 | 12,6 |
| Bt00007026 | CCNG1 | cyclin G1 | NM_001013364 | 1,97 | 6,94E-07 | 12,1 |
| Bt00003454 | BANK1 | B-cell scaffold protein with ankyrin repeats 1 | XM_002688119 | 1,78 | 7,36E-07 | 12,0 |
| Bt00004115 | HADH | hydroxyacyl-CoA dehydrogenase | NM_001046334 | 2,15 | 1,22E-06 | 11,4 |
| BLO_ext_01117 | HSD17B4 | hydroxysteroid (17-beta) dehydrogenase 4 | NM_001007809 | 2,39 | 1,59E-06 | 11,1 |
| BLO_ext_00320 | DYNLL1 | cytoplasmic dynein light polypeptide 1 | NM_001003901 | 2,02 | 1,97E-06 | 10,8 |
| Bt00006382 | SIRT5 | sirtuin 5 | NM_001034295 | 1,98 | 2,41E-06 | 10,6 |
| Bt00000583 | C1QBP | complement component 1, q subcomponent binding protein (C1QBP), nuclear gene encoding mitochondrial protein | NM_001034527 | 1,99 | 3,03E-06 | 10,3 |
| Bt00002141 | GMPS | guanine monphosphate synthetase | BC111273 | 1,77 | 4,24E-06 | 9,9 |
| Bt00003030 | HSD17B10 | hydroxysteroid (17-beta) dehydrogenase 10 | NM_174334 | 1,86 | 4,61E-06 | 9,8 |
| Bt00003407 | TRMT112 | tRNA methyltransferase 11-2 homolog (S. cerevisiae) | NM_001045981 | 1,85 | 4,61E-06 | 9,8 |
| Bt00004269 | SRSF9 | serine/arginine-rich splicing factor 9 | NM_001083398 | 1,77 | 5,91E-06 | 9,5 |
| BLO_ext_00630 | zfp106 | zinc finger protein 106 homolog (mouse) | XM_002690810 | 2,08 | 9,57E-06 | 8,9 |
| Bt00006196 | C12orf57 | chromosome 5 open reading frame, human C12orf57 | NM_001075525 | 1,79 | 1,16E-05 | 8,6 |
| BLO_ext_00470 | BCL11A | B-cell CLL/lymphoma 11A (zinc finger protein) | NM_001076121 | 1,95 | 1,24E-05 | 8,5 |
| Bt00003445 | TEP1 | telomerase-associated protein 1 | NM_001206912 | 2,27 | 1,47E-05 | 8,3 |
| Bt00007687 | MRPS18B | mitochondrial ribosomal protein S18B | NM_001038524 | 1,77 | 1,66E-05 | 8,1 |
| Bt00000174 | VBP1 | von Hippel-Lindau binding protein 1 | NM_001038516 | 1,71 | 1,69E-05 | 8,1 |
| Bt00007914 | PACSIN1 | protein kinase C and casein kinase substrate in neurons 1 | NM_001101101 | 2,42 | 1,90E-05 | 7,9 |
| BLO_ext_01382 | SYK | spleen tyrosine kinase | NM_001037465 | 1,75 | 2,13E-05 | 7,8 |
| Bt00003395 | PPP2CB | protein phosphatase 2, catalytic subunit, beta isozyme | NM_001075857 | 1,73 | 2,21E-05 | 7,7 |
| Bt00007822 | EEF1B2 | eukaryotic translation elongation factor 1 beta 2 | NM_001014936 | 1,95 | 2,36E-05 | 7,6 |
| BLO_ext_00531 | SORCS1 | sortilin-related VPS10 domain containing receptor 1 | NM_001191207 | 2,03 | 2,48E-05 | 7,5 |
| Bt00001201 | PHGDH | phosphoglycerate dehydrogenase | NM_001035017 | 1,64 | 3,17E-05 | 7,2 |
| Bt00006955 | GDI2 | GDP dissociation inhibitor 2 | NM_001033762 | 1,74 | 3,72E-05 | 7,1 |
| Bt00006263 | BCCIP | BRCA2 and CDKN1A interacting protein | NM_001046122 | 1,72 | 3,95E-05 | 7,0 |
| Bt00001656 | RTN4 | reticulon 4 | XM_005212571 | 1,55 | 3,98E-05 | 6,9 |
| Bt00004022 | NDUFB5 | NADH dehydrogenase (ubiquinone) 1 beta subcomplex, 5, 16kDa (NDUFB5), nuclear gene encoding mitochondrial protein | NM_176656 | 1,91 | 4,15E-05 | 6,9 |
| Bt00008119 | MAP4K3 | mitogen-activated protein kinase kinase kinase kinase 3 | NM_001191162 | 2,25 | 4,70E-05 | 6,8 |
| Bt00007285 | TCF4 | transcription factor 4 | NM_001034621 | 2,22 | 4,80E-05 | 6,7 |
| Bt00006771 | SDHA | succinate dehydrogenase complex, subunit A, flavoprotein | NM_174178 | 1,61 | 4,80E-05 | 6,7 |
| BLO_ext_00504 | WDFY4 | WDFY family member 4 | NM_001205945 | 1,87 | 4,80E-05 | 6,7 |
| Bt00007599 | ELOVL5 | ELOVL fatty acid elongase 5 | NM_001046597 | 2,04 | 6,06E-05 | 6,4 |
| Bt00006525 | NMI | N-myc (and STAT) interactor | NM_001035098 | 1,66 | 6,30E-05 | 6,4 |
| Bt00006958 | SYNGR2 | synaptogyrin 2 | NM_001100358 | 1,69 | 6,30E-05 | 6,3 |
| BLO_ext_00756 | CXXC5 | CXXC finger protein 5 | NM_001038176 | 2,36 | 6,56E-05 | 6,3 |
| Bt00008112 | SMIM20 | small integral membrane protein 20 | NM_001145428 | 1,65 | 7,33E-05 | 6,1 |
| Bt00008024 | HMGN1 | high mobility group nucleosome binding domain 1 | NM_001034772 | 1,72 | 7,50E-05 | 6,1 |
| Bt00007525 | LUZP6 | leucine zipper protein 6 | XM_005198116 | 1,86 | 7,50E-05 | 6,1 |
| Bt00002199 | FAM53B | family with sequence similarity 53, member B | - | 1,54 | 7,73E-05 | 6,0 |
| Bt00006193 | GLMN | glomulin, FKBP associated protein | NM_001192020 | 1,73 | 7,93E-05 | 6,0 |
| Bt00007508 | MAT2A | methionine adenosyltransferase II, alpha | NM_001101131 | 1,75 | 8,44E-05 | 5,9 |
| Bt00007230 | FCGR2B, CD32 | Fc fragment of IgG, low affinity IIb, receptor (CD32) | NM_174539 | 1,85 | 9,12E-05 | 5,8 |
| BLO_ext_01326 | SOX5 | SRY (sex determining region Y)-box 5 | NM_001083471 | 2,19 | 9,33E-05 | 5,8 |
| BLO_ext_00921 | SUMO2 | SMT3 suppressor of mif two 3 homolog 2 (yeast) | BT021041 | 1,54 | 9,54E-05 | 5,8 |
| Bt00004992 | MDH2 | malate dehydrogenase 2, NAD (mitochondrial) | NM_001013587 | 1,52 | 9,81E-05 | 5,7 |
| Bt00007614 | ACTL6A | actin-like 6A | NM_001105035 | 1,78 | 1,00E-04 | 5,7 |
| Bt00007141 | CYB5R3 | cytochrome b5 reductase 3 | NM_001103250 | 1,63 | 1,10E-04 | 5,5 |
| Bt00005626 | B3GNT5 | UDP-GlcNAc:betaGal beta-1,3-N-acetylglucosaminyltransferase 5 | NM_001076979 | 1,56 | 1,19E-04 | 5,5 |
| BLO_ext_01475 | CD79B | CD79b molecule, immunoglobulin-associated beta, transcript variant 2 | XM_586841 | 2,30 | 1,19E-04 | 5,4 |
| Bt00001902 | TMEM134 | transmembrane protein 134 | NM_001080254 | 1,65 | 1,60E-04 | 5,1 |
| Bt00000775 | SLC35F2 | solute carrier family 35, member F2 | XM_612258 | 1,82 | 1,60E-04 | 5,0 |
| Bt00000649 | PABPC4 | poly(A) binding protein, cytoplasmic 4 | XM_005204784 | 1,81 | 1,63E-04 | 5,0 |
| Bt00006645 | HSPA9 | heat shock 70kDa protein 9 (mortalin) | NM_001034524 | 1,54 | 1,70E-04 | 5,0 |
| Bt00007659 | DNASE2 | deoxyribonuclease II, lysosomal | NM_001075127 | 1,67 | 1,81E-04 | 4,9 |
| Bt00008210 | SNRPA1 | small nuclear ribonucleoprotein polypeptide A | NM_001098948 | 1,50 | 1,83E-04 | 4,9 |
| Bt00005583 | EIF4A2 | eukaryotic translation initiation factor 4A2 | NM_001034044 | 1,52 | 1,92E-04 | 4,8 |
| Bt00007483 | HAT1 | histone acetyltransferase 1 | NM_001034347 | 1,68 | 1,99E-04 | 4,8 |
| Bt00005950 | RHNO1 | chromosome 5 open reading frame, human C12orf32, RAD9-HUS1-RAD1 interacting nuclear orphan 1 | NM_001075631 | 1,81 | 2,00E-04 | 4,7 |
| Bt00007052 | STX7 | syntaxin 7 | NM_001077864 | 1,88 | 2,21E-04 | 4,6 |
| BLO_ext_00188 | SP100 | SP100 nuclear antigen | XM_001789440 | 1,66 | 2,21E-04 | 4,6 |
| Bt00005769 | MIEN1 | migration and invasion enhancer 1 | NM_001075221 | 1,67 | 2,21E-04 | 4,6 |
| TC69434 | RPL19 | ribosomal protein L19 | NM_001040516 | 1,60 | 2,21E-04 | 4,6 |
| Bt00003197 | ARPC5L | actin related protein 2/3 complex, subunit 5-like | NM_001034737 | 1,56 | 2,40E-04 | 4,5 |
| Bt00001990 | MRPL50 | mitochondrial ribosomal protein L50 | NM_001046180 | 1,65 | 2,42E-04 | 4,5 |
| Bt00007457 | MAT2B | methionine adenosyltransferase II, beta | NM_001046526 | 1,53 | 2,58E-04 | 4,4 |
| Bt00008239 | PAIP2 | poly(A) binding protein interacting protein 2 | NM_001034636 | 1,73 | 2,58E-04 | 4,4 |
| Bt00000171 | YWHAG | tyrosine 3-monooxygenase/tryptophan 5-monooxygenase activation protein, gamma polypeptide | NM_174793 | 1,57 | 2,70E-04 | 4,3 |
| Bt00001860 | THAP9 | THAP domain containing 9 | NM_001244183 | 1,88 | 2,92E-04 | 4,2 |
| BLO_ext_01202 | GLRX3 | glutaredoxin 3 | NM_001035101 | 1,58 | 3,02E-04 | 4,2 |
| Bt00007330 | TERF2 | telomeric repeat binding factor 2 | NM_001205589 | 1,72 | 3,49E-04 | 4,0 |
| Bt00007325 | TMBIM6 | transmembrane BAX inhibitor motif containing 6 | NM_001076414 | 1,64 | 3,57E-04 | 4,0 |
| BLO_ext_01034 | SYNCRIP | synaptotagmin binding, cytoplasmic RNA interacting protein | XM_589161 | 1,59 | 4,19E-04 | 3,8 |
| Bt00007267 | RBBP7 | retinoblastoma binding protein 7 | NM_001034638 | 1,61 | 4,67E-04 | 3,7 |
| Bt00001318 | LYN | v-yes-1 Yamaguchi sarcoma viral related oncogene homolog | NM_001177740 | 1,74 | 4,67E-04 | 3,7 |
| Bt00006534 | ADAM9 | ADAM metallopeptidase domain 9 | NM_001192818 | 2,11 | 5,45E-04 | 3,5 |
| BLO_ext_00292 | PEBP1 | phosphatidylethanolamine binding protein 1 | NM_001033623 | 1,90 | 5,69E-04 | 3,5 |
| Bt00006495 | C15H11orf31 | chromosome 15 open reading frame, human C11orf31 | NM_001164092 | 1,54 | 5,67E-04 | 3,5 |
| Bt00005180 | NAE1 | NEDD8 activating enzyme E1 subunit 1 | NM_001193033 | 1,57 | 5,74E-04 | 3,4 |
| Bt00002312 | TES | testis derived transcript (3 LIM domains) | XM_005205431 | 2,04 | 5,74E-04 | 3,4 |
| Bt00006613 | SEC31A | SEC31 homolog A (S. cerevisiae) | NM_001191274 | 1,59 | 5,83E-04 | 3,4 |
| Bt00007327 | GNL3 | guanine nucleotide binding protein-like 3 nucleolar | XM_005898122 | 1,50 | 6,01E-04 | 3,3 |
| Bt00006657 | CDCA7L | cell division cycle associated 7-like | NM_001104977 | 1,72 | 6,12E-04 | 3,3 |
| Bt00007278 | ATP5A1 | ATP synthase, H+ transporting, mitochondrial F1 complex, alpha subunit 1 | NM_174684 | 1,54 | 6,12E-04 | 3,3 |
| Bt00007668 | TPD52 | tumor protein D52 | NM_001076922 | 1,50 | 6,82E-04 | 3,2 |
| Bt00007561 | GMPS | guanine monphosphate synthetase | NM_001206436 | 1,78 | 7,01E-04 | 3,1 |
| Bt00003153 | DEXI | Dexi homolog (mouse) | NM_001191169 | 2,19 | 7,48E-04 | 3,0 |
| Bt00001138 | CABIN1 | calcineurin binding protein 1 | XM_002694694 | 1,73 | 8,08E-04 | 2,9 |
| Bt00006247 | PPM1E | protein phosphatase, Mg2+/Mn2+ dependent, 1E | XM_002695602 | 2,26 | 8,60E-04 | 2,9 |
| Bt00007674 | SNRPD3 | small nuclear ribonucleoprotein D3 polypeptide 18kDa | NM_001076479 | 1,50 | 8,74E-04 | 2,8 |
| Bt00007112 | EIF2S3 | eukaryotic translation initiation factor 2, subunit 3 gamma, 52kDa | NM_001046117 | 1,69 | 9,32E-04 | 2,8 |
| Bt00000308 | RRP7A | ribosomal RNA processing 7 homolog A (S. cerevisiae) | NM_001110074 | 1,62 | 9,45E-04 | 2,7 |
| Bt00001196 | ZCCHC11) | uridylyltransferase 4-like | NM_001206930 | 1,95 | 9,74E-04 | 2,7 |
| Bt00004145 | CXXC5 | CXXC finger protein 5 | NM_001038176 | 1,74 | 9,98E-04 | 2,7 |
| BLO_ext_00227 | CIITA | major histocompatibility complex class II transactivator | XM_002697962 | 1,71 | 1,04E-03 | 2,6 |
| Bt00001562 | ERH | enhancer of rudimentary homolog (Drosophila) | NM_001034336 | 1,57 | 1,06E-03 | 2,6 |
| BLO_ext_00586 | NIFK | MKI67 (FHA domain) interacting nucleolar phosphoprotein | NM_001034354 | 1,57 | 1,17E-03 | 2,5 |
| Bt00005854 | AVEN | Bos taurus apoptosis, caspase activation inhibitor | XM_002690781 | 1,84 | 1,21E-03 | 2,4 |
| BLO_ext_00441 | CMTM7 | CKLF-like MARVEL transmembrane domain containing 7 | NM_001075960 | 1,80 | 1,39E-03 | 2,3 |
| Bt00000168 | GPN3 | GPN-loop GTPase 3 | NM_001075272 | 1,58 | 1,44E-03 | 2,2 |
| Bt00006726 | RPF1 | ribosome production factor 1 homolog (S. cerevisiae) | NM_001083417 | 1,54 | 1,44E-03 | 2,2 |
| Bt00002187 | NACA | nascent polypeptide-associated complex alpha subunit | NM_001014916 | 1,52 | 1,50E-03 | 2,2 |
| Bt00001409 | DOB | major histocompatibility complex, class II, DO beta | NM_001013600 | 1,93 | 1,50E-03 | 2,1 |
| Bt00000851 | EHHADH | enoyl-CoA, hydratase/3-hydroxyacyl CoA dehydrogenase | NM_001075780 | 1,75 | 1,56E-03 | 2,1 |
| Bt00000901 | APIP | APAF1 interacting protein | NM_001075352 | 1,74 | 1,61E-03 | 2,1 |
| BLO_ext_01724 | HSPA14 | heat shock 70kDa protein 14 | NM_001046388 | 1,50 | 1,88E-03 | 1,9 |
| Bt00003631 | BMS1 | BMS1 homolog, ribosome assembly protein (yeast) | NM_001206147 | 1,56 | 2,06E-03 | 1,7 |
| BLO_ext_00097 | SPIB | Spi-B transcription factor (Spi-1/PU.1 related) | NM_001205444 | 1,79 | 2,25E-03 | 1,6 |
| Bt00003549 | HSD3B7 | hydroxy-delta-5-steroid dehydrogenase, 3 beta- and steroid delta-isomerase 7 | NM_001034696 | 1,61 | 2,28E-03 | 1,6 |
| Bt00007244 | ENOPH1 | enolase-phosphatase 1 | NM_001075875 | 1,53 | 2,70E-03 | 1,4 |
| BLO_ext_01824 | HSPA4 | heat shock 70kDa protein 4 | NM_001114192 | 1,69 | 2,70E-03 | 1,4 |
| Bt00003818 | JDP2 | Jun dimerization protein 2 | NM_001193245 | 1,52 | 2,71E-03 | 1,4 |
| Bt00003559 | IPO4 | importin 4 | NM_001083661 | 1,79 | 2,87E-03 | 1,3 |
| Bt00000212 | CTPS | CTP synthase | NM_001077858 | 1,55 | 2,92E-03 | 1,3 |
| BLO_ext_00226 | IFI30 | interferon, gamma-inducible protein 30 | NM_001101251 | 1,61 | 2,96E-03 | 1,3 |
| Bt00008064 | NCOA7 | nuclear receptor coactivator 7 | XM_003586458 | 1,71 | 3,00E-03 | 1,2 |
| Bt00007123 | NAT1 | N-acetyltransferase 1 (arylamine N-acetyltransferase) | NM_001075572 | 1,71 | 3,15E-03 | 1,2 |
| Bt00001848 | PIGO | phosphatidylinositol glycan anchor biosynthesis, class O | XM_001251645 | 1,51 | 3,25E-03 | 1,1 |
| BLO_ext_01021 | STARD7 | StAR-related lipid transfer (START) domain containing 7 | NM_001102086 | 1,62 | 3,28E-03 | 1,1 |
| BLO_ext_01667 | ACAT1 | acetyl-CoA acetyltransferase 1 | NM_001046075 | 1,56 | 3,28E-03 | 1,1 |
| Bt00000109 | CRTC3 | CREB regulated transcription coactivator 3 | XM_591546 | 1,57 | 3,39E-03 | 1,1 |
| Bt00005839 | TRMT5 | TRM5 tRNA methyltransferase 5 homolog (S. cerevisiae) | NM_001034565 | 1,58 | 3,52E-03 | 1,0 |
| Bt00007248 | PTCD3 | Pentatricopeptide repeat domain 3 | NM_001046034 | 1,61 | 3,78E-03 | 0,9 |
| Bt00000547 | CDC16 | cell division cycle 16 homolog (S. cerevisiae) | NM_001192990 | 1,55 | 4,04E-03 | 0,9 |
| Bt00007542 | RSRC1 | arginine/serine-rich coiled-coil 1 | NM_001075404 | 1,58 | 4,15E-03 | 0,8 |
| Bt00004188 | UBA2 | ubiquitin-like modifier activating enzyme 2 | NM_001083381 | 1,57 | 4,32E-03 | 0,8 |
| Bt00006536 | AP1B1 | adaptor-related protein complex 1, beta 1 subunit | NM_001045938 | 1,53 | 4,55E-03 | 0,7 |
| BLO_ext_01807 | LCN2 | lipocalin 2 | XM_005908492 | 2,38 | 4,83E-03 | 0,6 |
| Bt00000276 | ITPR1 | inositol 1,4,5-trisphosphate receptor, type 1 | NM_174841 | 1,57 | 5,15E-03 | 0,5 |
| Bt00008313 | HMGB1 | high mobility group box 1 | NM_176612 | 1,51 | 5,16E-03 | 0,5 |
| Bt00003457 | CFL2 | cofilin 2 | XM_005891814 | 1,87 | 5,61E-03 | 0,4 |
| BLO_ext_01003 | CXCL10 | chemokine (C-X-C motif) ligand 10 | NM_001046551 | 2,36 | 5,63E-03 | 0,4 |
| Bt00005244 | CBX6 | chromobox homolog 6 | NM_001103094 | 1,75 | 5,80E-03 | 0,4 |
| BLO_ext_01199 | KAT6B | K(lysine) acetyltransferase 6B | XM_005226509 | 1,68 | 6,13E-03 | 0,3 |
| Bt00001413 | DYNLL1 | dynein, light chain, LC8-type 1 | NM_001003901 | 1,56 | 6,60E-03 | 0,2 |
| Bt00005869 | CXHXorf57 | chromosome X open reading frame, human CXorf57 | - | 1,92 | 6,97E-03 | 0,2 |
| Bt00000303 | ZBTB32 | zinc finger and BTB domain containing 32 | NM_001191224 | 2,22 | 6,99E-03 | 0,2 |
| Bt00005707 | BBIP1 | BBSome interacting protein 1 | XM_003584100 | 1,57 | 7,17E-03 | 0,1 |
| Bt00002553 | TUBB4B | tubulin, beta 2C | NM_001034663 | 1,61 | 7,86E-03 | 0,0 |
| Bt00005269 | NR2F6 | nuclear receptor subfamily 2, group F, member 6 | XM_002704716 | 2,09 | 8,01E-03 | 0,0 |
| BLO_ext_01547 | ATM | ataxia telangiectasia mutated | NM_001205935 | 1,55 | 8,39E-03 | -0,1 |
| Bt00005894 | RBM34 | RNA binding motif protein 34 | NM_001110082 | 1,68 | 8,83E-03 | -0,2 |
| Bt00001001 | PKM | pyruvate kinase | NM_001205727 | 1,59 | 8,97E-03 | -0,2 |
| BLO_ext_00089 | SLC7A5 | solute carrier family 7 (amino acid transporter light chain, L system), member 5 | NM_174613 | 1,54 | 8,99E-03 | -0,2 |
| Bt00006287 | SNRNP70 | small nuclear ribonucleoprotein 70kDa (U1 | NM_001076046 | 1,53 | 9,32E-03 | -0,2 |
| Bt00004357 | ITPR3 | inositol 1,4,5-trisphosphate receptor, type 3 | NM_174370 | 1,56 | 9,32E-03 | -0,2 |
|  |  |  |  |  |  |  |
